# Role of sirtuin 1 in the brain development in congenital hypothyroidism rats via the regulation of p53 signaling pathway

**DOI:** 10.1080/21655979.2022.2060626

**Published:** 2022-04-06

**Authors:** Xiaofang Wei, Juan Tan, Hui Gao

**Affiliations:** a Department of Obstetrics, Lianyungang Maternal and Child Health Hospital, Lianyungang, Jiangsu, China; b Department of Genetics and Prenatal Diagnosis, Lianyungang Maternal and Child Health Hospital, Lianyungang, Jiangsu, China; c Department of Pediatrics, Lianyungang Affiliated Hospital Nanjing University of Traditional Chinese Medicine, Lianyungang, Jiangsu, China

**Keywords:** Congenital hypothyroidism, sirtuin 1, hippocampal neurons, p53 signaling pathway, apoptosis

## Abstract

The present study aimed to investigate the expression, role, and underlying mechanism of action of sirtuin 1 (SIRT1) in congenital hypothyroidism (CH). A CH model was established in rats, and neuronal cells were isolated from the hippocampal tissues of normal rats. Free thyroxine (fT4) and thyroid-Stimulating hormone (TSH) concentrations were determined to confirm CH model conduction. The cognitive behavior of rats with CH was examined using open field and forced swimming tests. Reverse transcription-quantitative polymerase chain reaction (RT-qPCR) and western blotting were used to detect the expression levels of SIRT1, p53, B-cell lymphoma-extra-large (Bcl-xl), Bcl-2-associated X (Bax), and cytochrome c in the hippocampal tissues and neuronal cells. The 3-(4,5-dimethylthiazol-2-yl)-2,5-diphenyl tetrazolium bromide assay and flow cytometry were performed to evaluate cell viability and apoptosis, respectively. The results revealed that SIRT1 was expressed at low levels in the hippocampal tissues of rats with CH. Moreover, overexpression of SIRT1 in the hippocampal tissues of rats with CH and improved rat behavior, while reducing the CH-induced nerve cell apoptosis. In addition, this overexpression increased the viability, inhibited apoptosis, and reduced the expression of p53, Bax, and cytochrome c, while increasing the expression of Bcl-xl in cultured neurons. In contrast, SIRT1-small interfering RNA exhibited the opposite effects in cultured neurons. In conclusion, SIRT1 plays a role in the occurrence and development of CH by regulating nerve cell apoptosis.

## Graphical abstract

**Figure:**
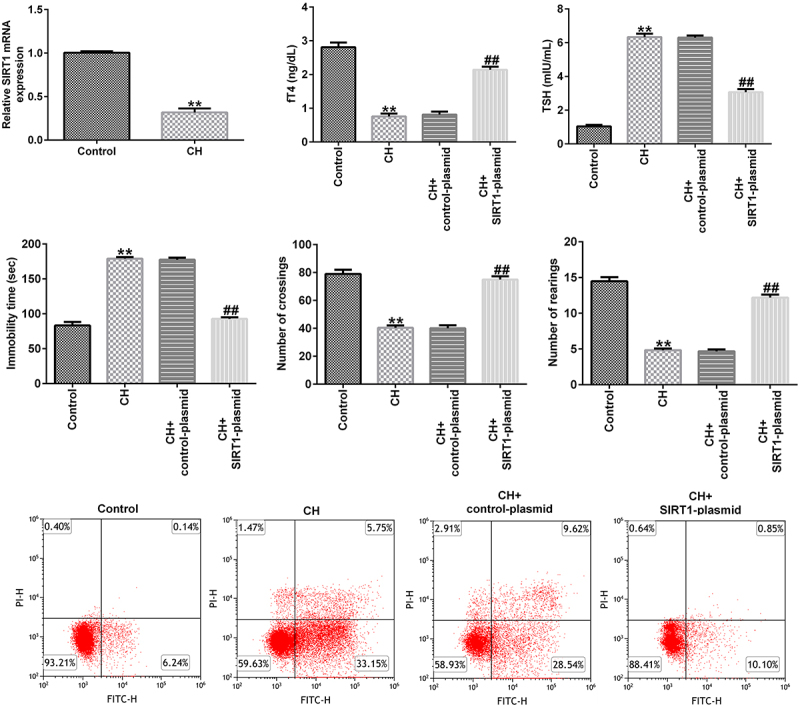

## Introduction

Congenital hypothyroidism (CH), caused by insufficient thyroid hormone synthesis or receptor defects, is a common endocrine disease in children [1,2]. Most patients present symptoms, such as intellectual disability, growth retardation, and low physical function in infancy. The national incidence rate of CH is 1/4,000 individuals, which is the highest among all neonatal diseases detected by screening [3,4]. Based on the cause, CH can be divided into sporadic hypothyroidism and endemic hypothyroidism [5]. Sporadic hypothyroidism is caused by the abnormal, absent, or ectopic thyroid tissue development during embryonic growth and enzyme defects in thyroid hormone synthesis (autosomal recessive inheritance). Endemic hypothyroidism is caused by a lack of iodine in the diet of pregnant women [6,7]. The incidence of endemic hypothyroidism has markedly decreased with the widespread use of iodized salt in China. Early diagnosis and treatment of the disease can facilitate normal development in affected children. Therefore, effective diagnostic methods for the assessment of the fetus with intrauterine growth retardation are important, and effective therapeutic strategy for CH are urgently needed.

The decreased or failed thyroid hormone synthesis is closely associated with the occurrence of CH [8]. Thyroid hormones are important for the development of the central nervous system, as they not only affect the development of the central nervous system, but also affect the activities of the differentiated and mature nervous systems [9,10]. In hyperthyroidism, the increased excitability of the central nervous system is mainly manifested as difficulty in concentrating, allergic concerns, sentimentality, mood disorders, restlessness, poor sleep, and muscle fibrillation [11,12]. In contrast, when thyroid function is low, the excitability of the central nervous system is reduced, memory loss occurs, speech and movement are slow, and apathy and sleepiness occur throughout the entire day [13]. The hippocampus is an important organ in the central nervous system and participates in learning and memory storage [14]. Therefore, the hippocampus has become the focus of numerous studies on the pathogenesis of CH.

Sirtuin 1 (SIRT1) is a nicotinamide-dependent protein deacetylase that promotes cell proliferation, invasion and angiogenesis [15–17]. However, to the best of our knowledge, there have been no published studies on the function of SIRT1 in CH.

In the present study, we hypothesized that SIRT1 plays an important role in CH by regulating the growth of hippocampal neurons. Therefore, in this study, we aimed to explore the specific role and related molecular mechanisms of SIRT1 in CH, which would provide an additional theoretical basis for the treatment of CH and facilitate the development of novel and effective congenital thyroid function targets for the diagnosis and treatment of hypothyroidism.

## Materials and methods

### Animals

Forty female pregnant Sprague Dawley rats (weight, 200 ± 5 g; age, 4–6 weeks) were obtained from the Experimental Animal Center of Shanghai (Shanghai, China) and housed in a controlled environment (temperature, 22 ± 1°C; humidity, 50–60%; 12-h light/dark cycle). All animal experiments were performed in accordance with the guidelines provided by the National Institutes of Health for the Care and Use of Laboratory Animals. The study protocol was approved by the Animal Ethics Committee of the Experimental Animal Center of the Lianyungang Maternal and Child Health Hospital (Lianyungang, China; approval number: LYG-MER2021045).

### Establishment of a rat CH model

To establish a CH rat model, pregnant rats were intraperitoneally injected with propylthiouracil (50 mg/day) on gestational day 15 and daily thereafter until parturition [18]. For CH therapy, 12-day-old newborn rats were anesthetized with 2% pentobarbital sodium (40 mg/kg; intraperitoneal injection), and their skulls were opened as previously described. Subsequently, the Control CRISPR Activation Plasmid (control plasmid, cat. no. sc-437,275; Santa Cruz Biotechnology, Inc.; 5 µl; 1 nmol/l) or SIRT1CRISPR Activation Plasmid (SIRT1-plasmid, cat. no. sc-400,085-ACT; Santa Cruz Biotechnology, Inc.; 5 µl; 1 nmol/l) was injected into the left lateral ventricle of 12-day-old rats using micro-syringes. Newborn rats were divided into the following groups (n = 10): i) control, ii) CH, iii) CH + control-plasmid, and iv) CH + SIRT1-plasmid. On day 21 after birth, the rats (body weight, <200 g) were anesthetized with pentobarbital sodium (40 mg/kg; intraperitoneal injection) and sacrificed by cervical dislocation. Death was verified by observing cardiac and respiratory arrest. Brain hippocampal tissues from different groups were obtained after euthanasia. The experiments were terminated when the rats lost >15% of their body weight prior to the injection. None of the rats died during the study period.

### Detection of plasma fT4 and TSH concentrations

The plasma from rat pup blood samples were collected through centrifugation (3500 rpm; 4°C; 15 min). Subsequently, the plasma was aliquoted and immediately frozen at −80°C (Wisecryo, Korea). Free T4 (fT4) levels were analyzed using immunoassays (Cobas, e601,Roche Diagnostics) according to the manufacturer’s instructions. The quantitative sandwich ELISA technique was used to determine plasma thyroid-Stimulating hormone (TSH) [19].

### Behavioral tests in rats

Behavioral testing, including open-field and forced swimming tests, was performed on rats with CH [19].

For the open field test, rats were first evaluated in an open field. The test was performed in a box that was divided into 16 small squares. Each rat was placed in a corner of the box and allowed to explore the site for 3 min, after which the number of crossings and rearings was recorded.

In the forced swimming test, the rats were placed in a cylindrical water bucket (25°C) for observation. At the end of every 5-second period for a total of 300 s, the frequency of various behaviors of rats, including climbing, standing still, and swimming, was recorded.

### Cell culture and transfection

Neuronal cells were isolated from the hippocampal tissues of normal rats. Briefly, after the rats were sacrificed, the hippocampal tissues were removed and aseptically cut into pieces, and the cells were separated by trypsin (0.25%; Sigma Aldrich, St. Louis, MO, USA) digestion, followed by culture in DMEM/F12 (Gibco; Thermo Fisher Scientific, Inc.) containing 20% fetal bovine serum (Gibco; Thermo Fisher Scientific, Inc.).

Neuronal cells were seeded in 6-well plates (5 × 10^4^ cells per well), allowed to grow overnight, and then transfected with 1 µg control-plasmid (cat. no. sc-437,275; Santa Cruz Biotechnology, Inc., 1 µg SIRT1-plasmid (cat. no. sc-437,275; Santa Cruz Biotechnology, Inc.), 0.2 µM control-small interfering RNA (siRNA; cat. no. sc-36,869; Santa Cruz Biotechnology, Inc.) or 0.2 µM SIRT1-siRNA (cat. no. sc-40,986; Santa Cruz Biotechnology, Inc.) using Lipofectamine® 2000 reagent (Invitrogen; Thermo Fisher Scientific, Inc.). After 48 h of transfection at 37°C, the cells were collected and the transfection efficiency was determined using reverse transcription-quantitative polymerase chain reaction (RT-qPCR) and western blotting.

### RNA extraction and RT-qPCR

Total RNA was obtained from the hippocampal tissues or neuronal cells using TRIzol® reagent (Invitrogen; Thermo Fisher Scientific, Inc.) and reverse-transcribed into cDNA using a cDNA synthesis kit (Invitrogen; Thermo Fisher Scientific, Inc.). All reactions were performed using a Prism 7000 Real-Time PCR System with SYBR qPCR Master Mix (Vazyme Biotech Co., Ltd.) to quantify the expression of genes. Primers were designed using the GenScript software. The amplification conditions for qPCR were as follows: initial denaturation at 95°C for 5 min, followed by 40 cycles of 10s at 95°C and 30s at 60°C. β-actin was used as an internal control. The relative mRNA expression levels of SIRT1, p53, B-cell lymphoma-extra-large (Bcl-xl), Bcl-2-associated X (Bax), and cytochrome c were calculated using the 2^−ΔΔCq^ method [20]. Primer sequences were listed as following:

SIRT1-forward, 5ʹ-TTCCAGCCATCTCTCTGTC-3ʹ;

reverse, 5ʹ-ATTCCCGCAACCTGTTC-3ʹ;

p53-forward, 5ʹ-CAGATCCTAGCGTCGAGCCCC-3ʹ;

reverse, 5ʹ-CTGGGTCTTCAGTGAACCATTGTTC-3ʹ;

Bcl-xl-forward, 5′-TGCAGGTATTGGTGAGTCGG-3′;

reverse, 5′-AAGCGTTCCTGGCCCTTTC-3′;

Bax-forward, 5ʹ-CCCGAGAGGTCTTTTTCCGAG-3ʹ;

reverse, 5ʹ-CCAGCCCATGATGGTTCTGAT-3ʹ;

cytochrome c-forward, 5′‐TTGCACTTACACCGGTACTTAAGC‐3ʹ;

reverse, 5′‐ACGTCCCCACTCTCTAAGTCCAA‐3′;

β-actin forward, 5′-GAGCACAGAGCCTCGCCTTT-3′;

reverse, 5′-GCCCACATAGGAATCCTTCTG-3′.

### Western blotting analysis

Neuronal cells or rat hippocampal tissues were lysed with the radioimmunoprecipitation assay buffer, and the lysate was centrifuged at 10,000 × g, at 4°C for 15 min to obtain the total protein. Next, the protein was quantified using a BCA protein assay kit (Beyotime Institute of Biotechnology), separated by 10% sodium dodecyl sulfate-polyacrylamide gel electrophoresis, and transferred onto a polyvinylidene fluoride membrane (EMD Millipore, Billerica, MA, USA), which was blocked with PBS-0.1%Tween 20 (PBST) containing 5% skimmed milk for 1 h. Subsequently, the membrane was incubated with antibodies against SIRT1 (cat. no. 2496; dilution 1: 1000; Cell Signaling Technology, Inc.), p53 (cat. no. 2527; dilution 1: 1000; Cell Signaling Technology, Inc.), Bcl-xl (cat. no. 2764; dilution 1: 1000; Cell Signaling Technology, Inc.), Bax (cat. no. 5023; dilution 1: 1000; Cell Signaling Technology, Inc.), cytochrome c (cat. no. 4280; dilution 1: 1000; Cell Signaling Technology, Inc.), or GAPDH (cat. no. 5174; dilution 1: 1000; Cell Signaling Technology, Inc.) at 4°C overnight. Next, the membrane was washed thrice with PBST and incubated with a secondary antibody (ab7090, 1:1000, Abcam) for 1 h at room temperature. The composition of the buffer used to dilute primary and secondary antibodies was 1× TBST with 5% BSA. Protein bands were visualized using an enhanced chemiluminescence substrate (Pierce; Thermo Fisher Scientific, Inc.), according to the manufacturer’s instructions [21]. GAPDH served as a loading control.

### Caspase 3 activity assay

The activity of caspase 3 was measured using a colorimetric assay kit (Beyotime Institute of Biotechnology) according to the manufacturer’s instructions [22]. Briefly, cells were collected by centrifugation at 600 × g at 4°C for 5 min, then lysed in an ice bath for 15 min. Subsequently, the cell lysates were centrifuged at 16,000 × g for 10 min at 4°C and the supernatant were transferred to an ice-cooled centrifuge tube. Finally, the enzymatic activity of caspase 3 was measured immediately.

### Flow cytometric (FCM) analysis

FCM analysis was performed to determine apoptosis [23]. Single-cell suspensions prepared from rat hippocampal tissues or transfected cells were collected. To obtain single-cell suspension from rat hippocampal tissues, 3 mm^3^ fresh cerebral cortex was digested with 0.25% trypsin (1 ml) without ethylene diamine tetraacetic acid for 30 min. The collected cells were then centrifuged at 1000 × g for 5 min, the supernatant was discarded, the cells were collected, and the cells were gently resuspended in PBS and counted. Subsequently, 100 µL of cell suspension (10^6^ cells) was incubated with 5 µL annexin V-fluorescein isothiocyanate (FITC) and propidium iodide (BD Biosciences), according to the manufacturer’s protocol. The stained cells were analyzed using a FACSCalibur flow cytometer (BD Biosciences), and the data were analyzed using the Kaluza analysis software (version 2.1.1.20653; Beckman Coulter, Inc.).

### 3-(4,5-dimethylthiazol-2-yl)-2,5-diphenyl tetrazolium bromide (MTT) assay

The MTT assay was used to assess the viability of neuronal cells [24]. After 48 h of transfection, the cells (1 × 10^4^ cells/well) were seeded into a 96-well plate, and 24 h after cell seeding, 20 μL of MTT solution (Beyotime Institute of Biotechnology) was added to each well and incubated for 4 h. After removing the medium, 100 μL dimethyl sulfoxide was added to each well to terminate the reaction. The absorbance was recorded at 570 nm using a microplate reader (Bio-Rad Laboratories, Inc.).

### Statistical analysis

Data are presented as the mean ± standard deviation of three independent experiments. Comparisons between groups were analyzed using the Student’s *t*-test or one-way analysis of variance (ANOVA). P < 0.05 was considered to indicate a statistically significant difference.

## Results

### SIRT1 expression levels in CH model rats

**Figure 1.**
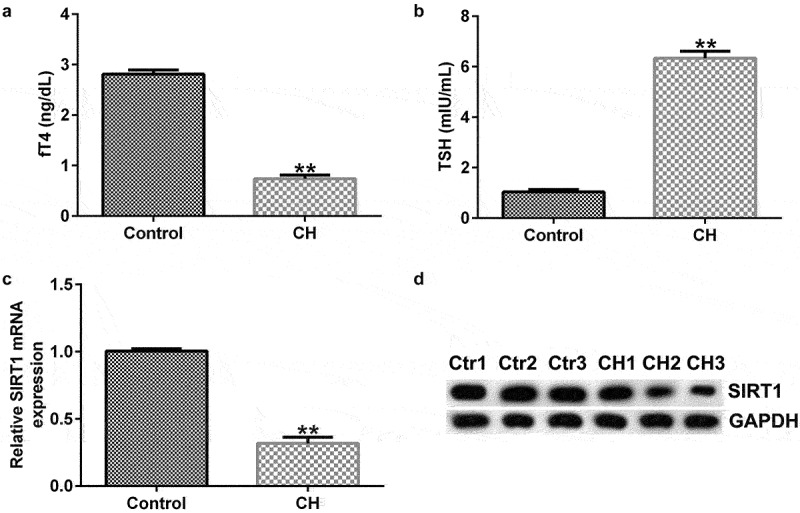
Expression levels of sirtuin 1 (SIRT1) in congenital hypothyroidism (CH) model rats. CH rat models were established with propylthiouracil. (a) fT4 level in the plasma of rats. (b) TSH level in the plasma of rats. (c) Reverse transcription-quantitative polymerase chain reaction (RT-qPCR) was performed to determine the mRNA expression levels of SIRT1 in the hippocampal tissues of CH and normal rats. **P < 0.01. (d) Protein expression levels of SIRT1 in rat hippocampal tissues were detected using by blotting analysis. SIRT1, sirtuin 1; CH, congenital hypothyroidism.

To confirm whether CH was successfully conducted, plasma concentration of free thyroid hormones such as fT4 and TSH were determined. As shown in Figure 1(a, b), compared with the control group, the fT4 level significantly reduced and the level of TSH enhanced, indicating the successful conduction of CH. To determine the expression of SIRT1 in CH model, the mRNA and protein expression levels of SIRT1 in the hippocampal tissues of normal and CH model rats were detected by RT-qPCR and western blotting, respectively. The results showed that the mRNA and protein expression levels of SIRT1 in the model group were significantly lower than those in the control group (Figure 1c, d).

### Protective effect of SIRT1 overexpression in CH model rats

**Figure 2.**
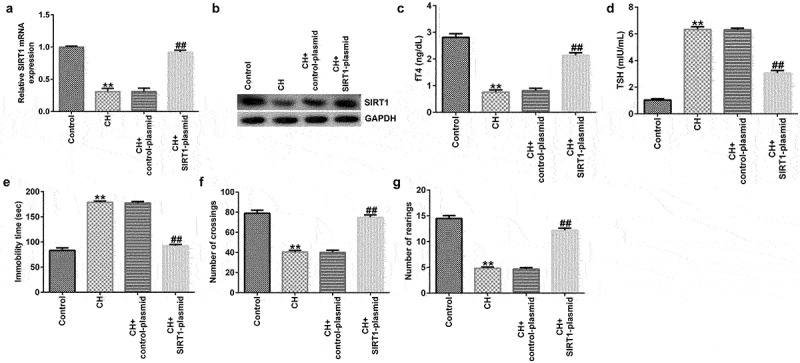
Effects of SIRT1-plasmid in CH model rats. (a) mRNA and (b) protein expression levels of SIRT1 in the hippocampal tissues of different groups of rats were determined using RT-qPCR and western blotting analyses, respectively. (c and d) fT4 and TSH levels in the plasma of rats. (e-g) Cognitive behavioral examination of rats with CH was performed via open-field and forced swimming tests. **P < 0.01 vs. control; ^##^P < 0.01 vs. CH + control-plasmid. SIRT1, sirtuin 1; CH, congenital hypothyroidism.

To explore the effects of SIRT1 in CH model rats, a SIRT1-plasmid was used to treat the rats, and the rats were divided into the following four groups: i) control, ii) CH, iii) CH + control-plasmid, and iv) CH + SIRT1-plasmid groups. As shown in Figure 2(a, b), compared with that of the control group, the expression of SIRT1 in the CH group was significantly reduced, and this reduction was significantly reversed by upon SIRT1 overexpression.

Besides, we found that compared to CH model group, SIRT1 overexpression significantly enhanced fT4 levels and reduced TSH levels in the plasma of CH rats (Figure 2(c, d)).

**Figure 3.**
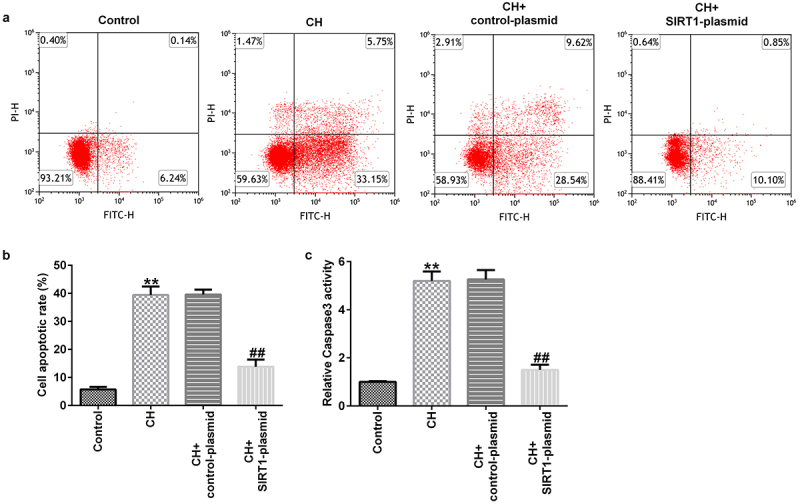
Effects of SIRT1-plasmid on hippocampal neuronal apoptosis in CH model rats.(a) Flow cytometry analysis was used to detect the apoptosis of rat hippocampal neurons. (b) Statistical graph of apoptosis rate. (c) Caspase 3 activity was determined using Caspase 3 activity detection kit. **P < 0.01 vs. control; ^##^P < 0.01 vs. CH + control-plasmid. SIRT1, sirtuin 1; CH, congenital hypothyroidism.

In addition, the results of the open-field and forced swimming tests indicated that SIRT1 overexpression improved the behavior of the CH model rats (Figure 2(e–g)). Furthermore, FCM results showed that SIRT1 overexpression reduced the CH-induced nerve cell apoptosis (Figure 3(a, b)) and caspase 3 activity (Figure 3(c)).

### Effect of SIRT1 overexpression on the apoptosis of cultured neurons

**Figure 4.**
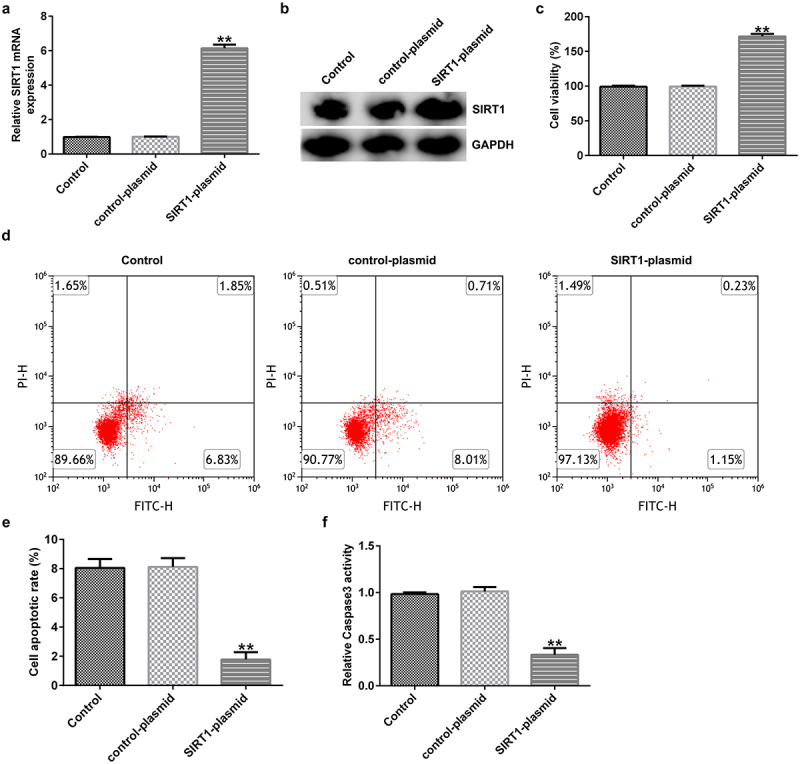
Effects of SIRT1-plasmid on the apoptosis of cultured neurons. (a) mRNA and (b) protein expression levels of SIRT1 in the transfected cells were evaluated using western blotting and RT-qPCR analyses, respectively. (c) Viability of the transfected cells was detected using the 3-(4,5-dimethylthiazol-2-yl)-2,5-diphenyl tetrazolium bromide (MTT) assay. (d and e) Flow cytometry analysis was used to detect the apoptosis of transfected cells. (f) Caspase 3 activity was determined. **P < 0.01 vs. control-plasmid. SIRT1, silent mating type information regulation 1/sirtuin 1.

Evaluation of the transfection efficiency in neuronal cells – isolated and cultured from normal rat hippocampal tissues – revealed that SIRT1 expression was significantly increased at the mRNA and protein levels upon transfecting with the SIRT1-plasmid for 48 h (Figure 4(a, b)). MTT assay and FCM analysis showed that SIRT1 overexpression remarkably increased the viability (Figure 4(c)) and inhibited apoptosis (Figure 4(d, e)) of cultured neurons. Compared with the control plasmid group, caspase 3 activity was significantly reduced in SIRT1-plasmid-transfected group (figure 4(f)).

### Effects of SIRT1 overexpression on the levels of the p53 signaling pathway related molecules

**Figure 5.**
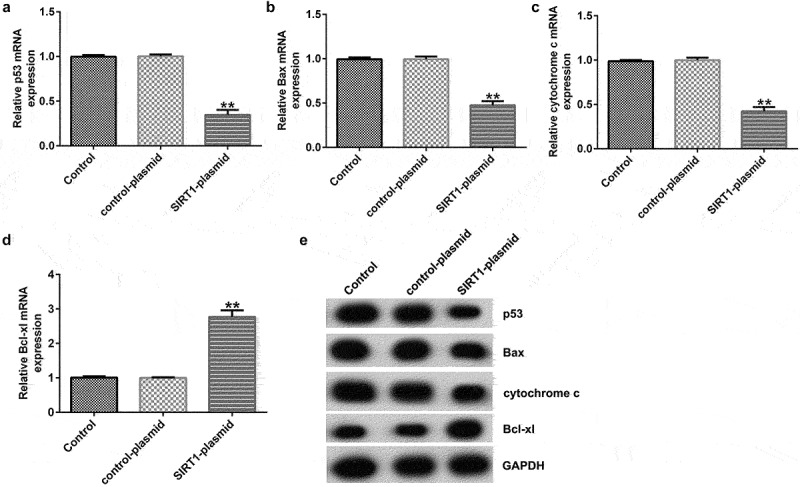
Effects of SIRT1-plasmid on the p53 signaling pathway in cultured neurons. (a-d) mRNA expression levels of p53, Bcl-xl, Bax, and cytochrome c in the transfected neurons were determined using RT-qPCR analysis. (e) Protein expression levels of p53, B-cell lymphoma-extra-large (Bcl-xl), Bcl-2-associated X (Bax), and cytochrome c in the transfected neurons were detected using western blotting analysis. **P < 0.01 vs. control-plasmid.

To further explore the mechanism of action of SIRT1 overexpression in cultured neurons, RT-qPCR and western blotting were performed to detect the expression of the p53 signaling pathway-related molecules (p53, Bcl-xl, Bax, and cytochrome c). The results showed that SIRT1 overexpression resulted in markedly reduced mRNA and protein expression of p53, Bax, and cytochrome c, and increased expression of Bcl-xl (Figure 5(a-e)) in cultured neurons.

### Effect of SIRT1 knockdown on the apoptosis of cultured neurons

**Figure 6.**
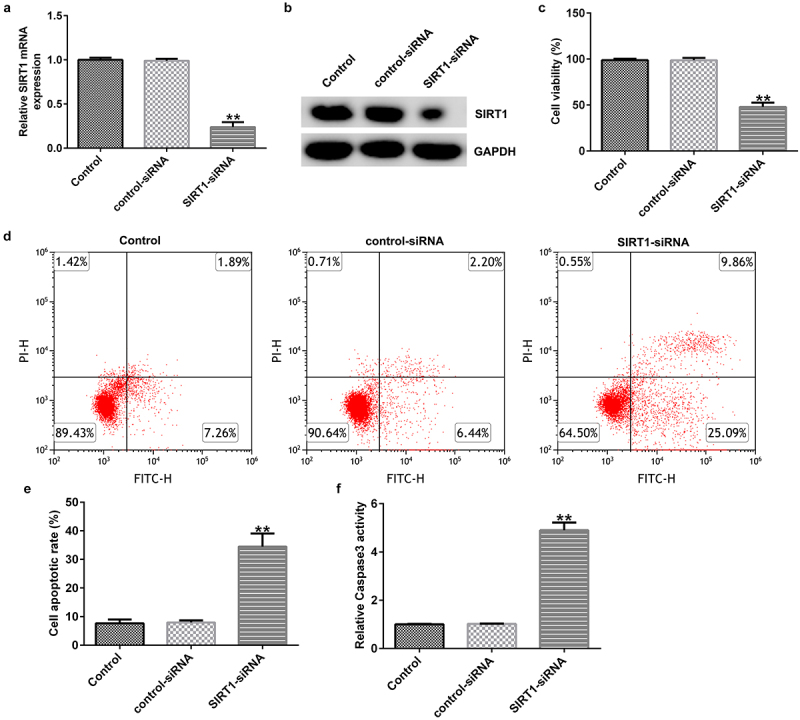
Effect of SIRT1-small interfering RNA (siRNA) on the apoptosis of cultured neurons. (a) mRNAexpression levels of SIRT1 in the transfected cells were evaluated using western blotting analysis. (b) Protein expression levels of SIRT1 in the transfected cells were determined using RT-qPCR analysis. (c) Viability of the transfected cells was detected using an MTT assay. (d and e) Flow cytometry analysis was used to detect the apoptosis of transfected cells. (f) Caspase 3 activity was determined. **P < 0.01 vs. control-siRNA. SIRT1, sirtuin 1; siRNA, small interfering RNA.

Evaluation of the transfection efficiency revealed that SIRT1-siRNA markedly reduced the expression of SIRT1 at mRNA and protein levels upon being transfected in cultured neurons for 48 h (Figure 6(a, b)). MTT assay and FCM analysis showed that SIRT1 knockdown significantly reduced the viability (Figure 6(c)) and promoted the apoptosis (Figure 6(d, e)) of cultured neurons. In addition, compared with the control siRNA group, caspase 3 activity was significantly enhanced in the SIRT1-siRNA-transfected group (figure 6(f)).

### Effects of SIRT1 knockdown on the levels of the p53 signaling pathway-related molecules in cultured neurons

**Figure 7.**
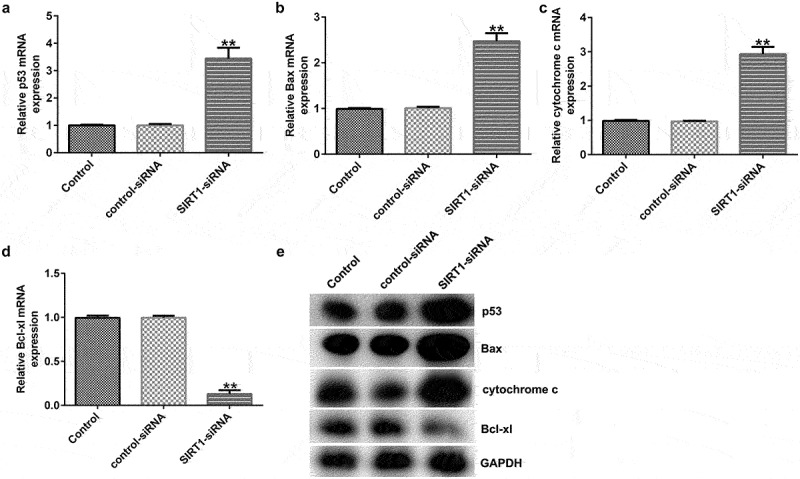
Effect of SIRT1-siRNA on the p53 signaling pathway in cultured neurons. (a-d) mRNA expression levels of p53, Bcl-xl, Bax, and cytochrome c in the transfected neurons were determined using RT-qPCR analysis. (e) Protein expression levels of p53, Bcl-xl, Bax, and cytochrome c in the transfected neurons were detected using western blotting analysis.**P < 0.01 vs. control-siRNA. siRNA, small interfering RNA.

To analyze the effects of SIRT1 knockdown on the p53 signaling pathway, RT-qPCR and western blotting showed that SIRT1 knockdown resulted in enhanced the expression of p53, Bax, and cytochrome c, and decreased the expression of Bcl-xl (Figure 7(a-e)) at mRNA and protein levels in cultured neurons.

## Discussion

CH, a common endocrine disease in children, is caused by receptor defects or iodine deficiency in the maternal diet during pregnancy [25]. CH is of two types, i.e., sporadic hypothyroidism and endemic hypothyroidism. The main clinical manifestations are physical and intellectual developmental disorders. The damage caused by CH in children is mainly due to abnormal brain development, which leads to intellectual disability in the affected children [26].

SIRT1, which is located on human chromosome 10q21.3, is expressed in developing and adult mammalian brains [27,28]. It has been reported that SIRT1 plays an important role in the occurrence and development of neurons, the maintenance of normal nerve function, and the protection of neurons [29]. Previous studies have shown that SIRT1 can regulate disease development by regulating nerve cell apoptosis [30,31]. However, to the best of our knowledge, there are no reports on the effects of SIRT1 on CH. In the present study, a CH model was established in rats using previously described methods. It was found that SIRT1 was significantly expressed at low levels in the hippocampal tissues of CH rats, and it was hypothesized that SIRT1 is involved in the occurrence and development of CH. In addition, a SIRT1-plasmid was used to treat rats with CH, and the results indicated that the SIRT1-plasmid could increase SIRT1 expression in the CH rats, enhance fT4 levels and reduce TSH levels in the plasma of CH rats, improve rat behavior, and reduce CH-induced nerve cell apoptosis.

These results indicate that SIRT1 participates in CH by regulating the apoptosis of nerve cells. Therefore, several *in vitro* experiments were performed to verify the effects of SIRT1 on cultured neurons and the associated mechanism of action was also evaluated. The results showed that the SIRT1-plasmid-induced SIRT1 overexpression resulted in increased viability of the neurons, and inhibited their apoptosis.

Previous studies have shown that the p53 signaling pathway plays a vital role in processes such as apoptosis and cell cycle regulation [32,33]. Current evidence suggests that p53 encodes a sequence-specific transcription factor that controls the expression of genes whose products (such as Bax; PUMA,p53-upregulated modulator of apoptosis) mediate apoptosis [34,35]. In addition, endogenous mitochondrial p53 forms inhibitory complexes with protective Bcl-xl and Bcl-2 proteins, leading to mitochondrial release of cytochrome c, suggesting that p53 can promote apoptosis by signaling directly on mitochondria [36]. Recent findings suggest that p53 is closely related to neuronal death [37,38]. In the present study, we detected the expression of molecules associated with the p53 signaling pathway in the background of SIRT1 overexpression and knockdown. The results showed that the SIRT1 overexpression reduced the expression of p53, Bax, and cytochrome c, and increased the expression of Bcl-xl in cultured neurons. Opposite results were obtained upon SIRT1 knockdown. The findings indicated that SIRT1 overexpression inhibited neuronal apoptosis via inhibiting p53 signaling pathway.

## Research highlights

- SIRT1 expression levels is significantly down-regulated in CH model rats;
- Protective effect of SIRT1 overexpression in CH model rats;
- SIRT1 overexpression inhibited the p53 signaling pathway in cultured neurons.

## Conclusion

The present study found that SIRT1 plays an important role in the progression of CH and is involved in its occurrence and development by regulating the apoptosis of nerve cells. These findings suggest that targeting SIRT1 may be a novel strategy for the clinical treatment of CH.

## Supplementary Material

Supplemental Material

## Data Availability

The datasets used and/or analyzed during the present study are available from the corresponding author upon reasonable request.
