# Supplementary figures and images for: Role of sirtuin 1 in the brain development in congenital hypothyroidism rats via the regulation of p53 signaling pathway
Source: Bioengineered. 2022 Apr 6;13(4):9455–66. doi: 10.1080/21655979.2022.2060626 (PMC9161855; doi:10.1080/21655979.2022.2060626)

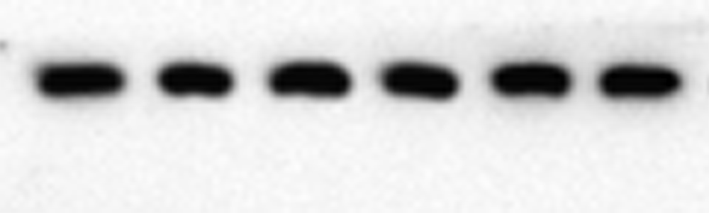

Supplement: Supplemental Material [file KBIE_A_2060626_SM1614.zip › supplementary materials/Original blots of western blot assay/Figure1/GAPDH.tif]

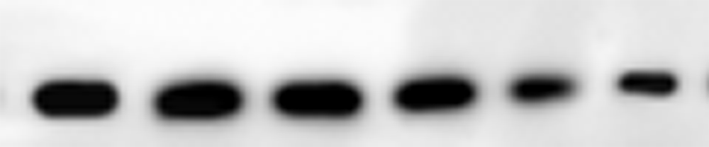

Supplement: Supplemental Material [file KBIE_A_2060626_SM1614.zip › supplementary materials/Original blots of western blot assay/Figure1/SIRT1.tif]

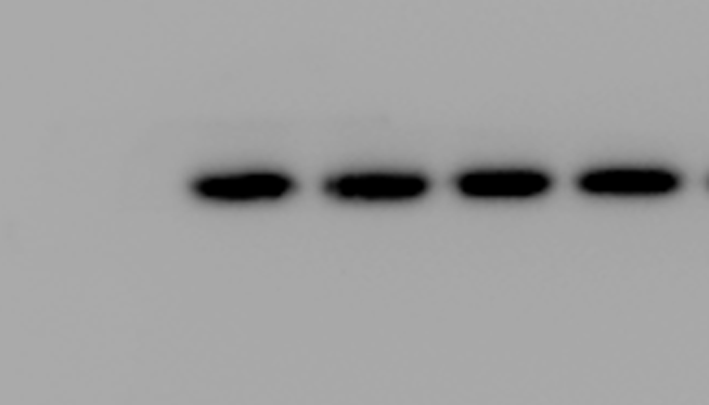

Supplement: Supplemental Material [file KBIE_A_2060626_SM1614.zip › supplementary materials/Original blots of western blot assay/Figure2/GAPDH.tif]

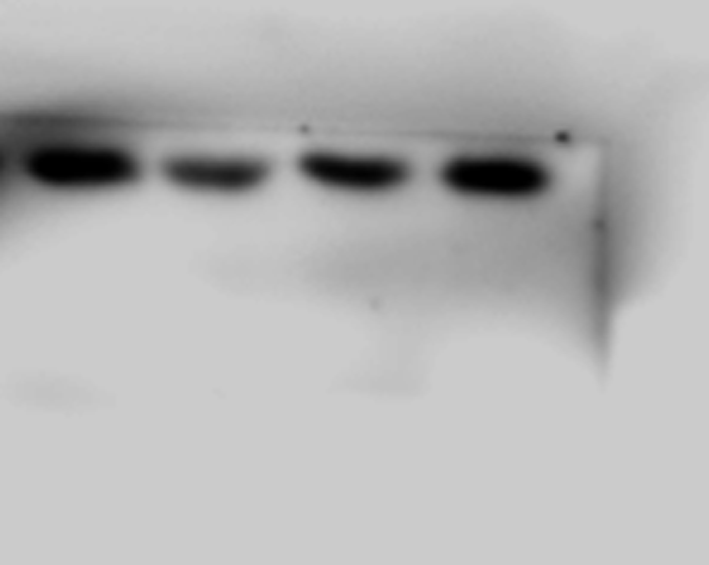

Supplement: Supplemental Material [file KBIE_A_2060626_SM1614.zip › supplementary materials/Original blots of western blot assay/Figure2/SIRT1.tif]

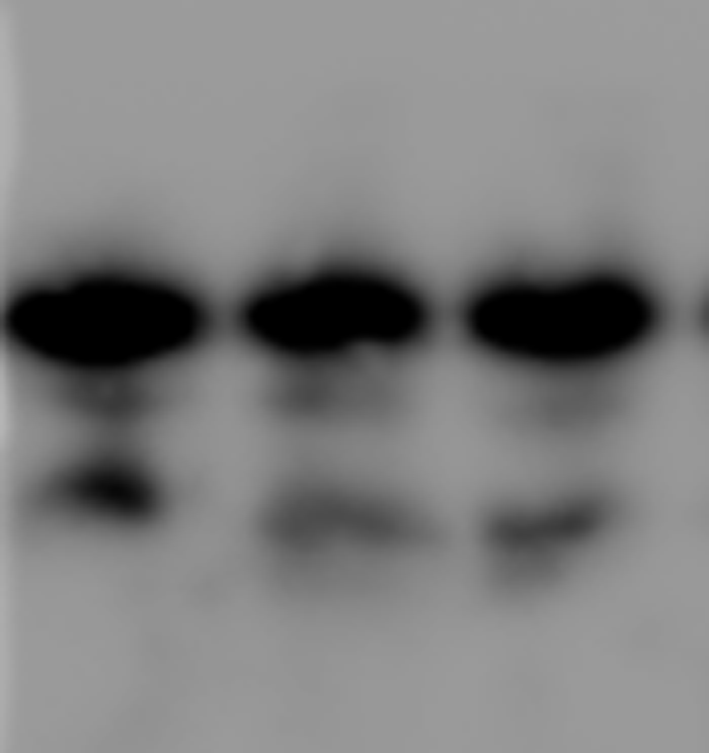

Supplement: Supplemental Material [file KBIE_A_2060626_SM1614.zip › supplementary materials/Original blots of western blot assay/Figure4/GAPDH.tif]

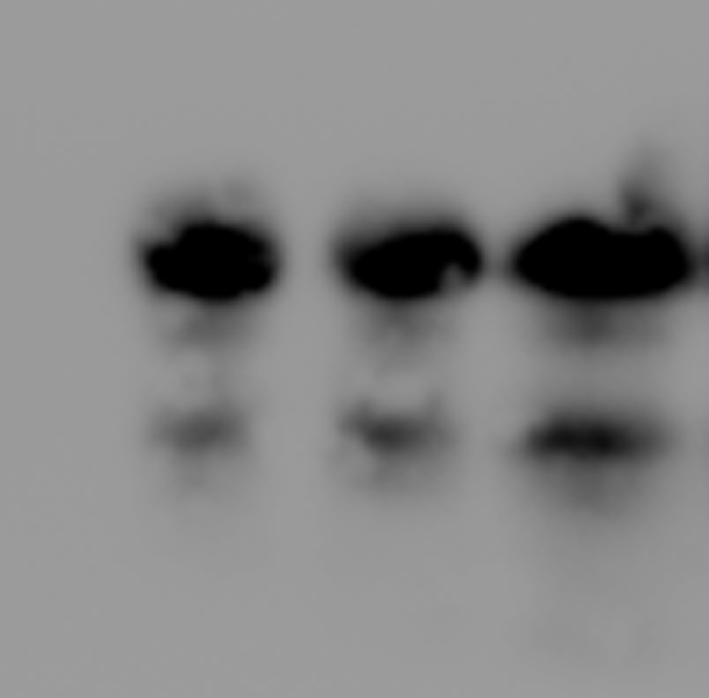

Supplement: Supplemental Material [file KBIE_A_2060626_SM1614.zip › supplementary materials/Original blots of western blot assay/Figure4/SIRT1.tif]

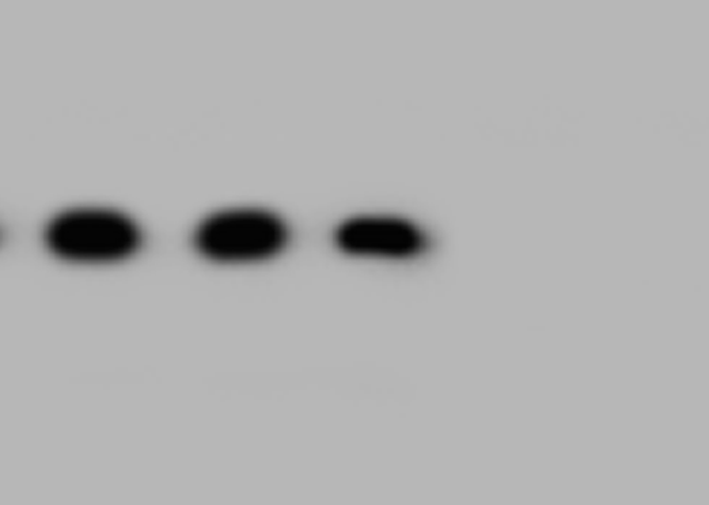

Supplement: Supplemental Material [file KBIE_A_2060626_SM1614.zip › supplementary materials/Original blots of western blot assay/Figure5/Bax.tif]

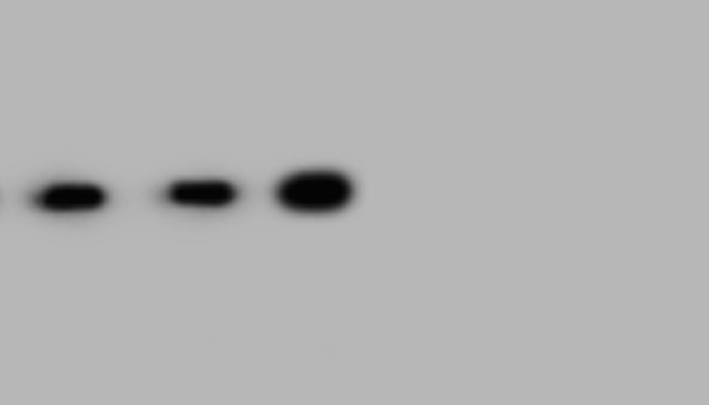

Supplement: Supplemental Material [file KBIE_A_2060626_SM1614.zip › supplementary materials/Original blots of western blot assay/Figure5/Bcl-xl.tif]

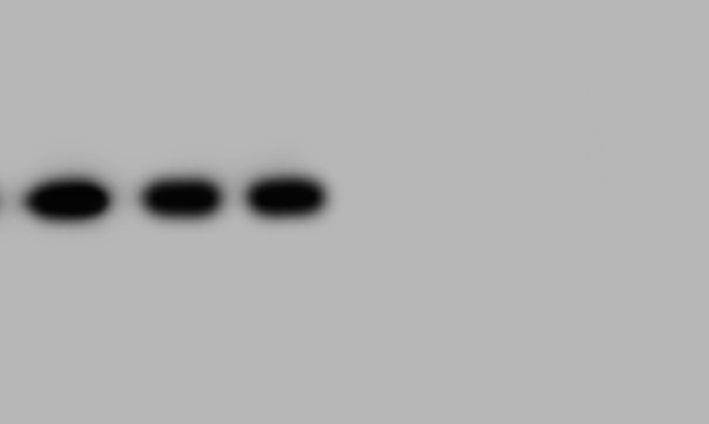

Supplement: Supplemental Material [file KBIE_A_2060626_SM1614.zip › supplementary materials/Original blots of western blot assay/Figure5/GAPDH.tif]

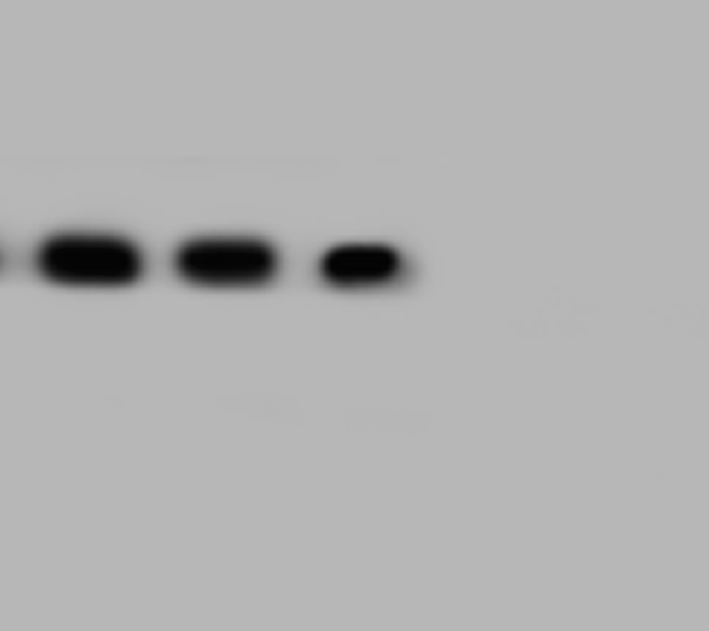

Supplement: Supplemental Material [file KBIE_A_2060626_SM1614.zip › supplementary materials/Original blots of western blot assay/Figure5/cytochrome c.tif]

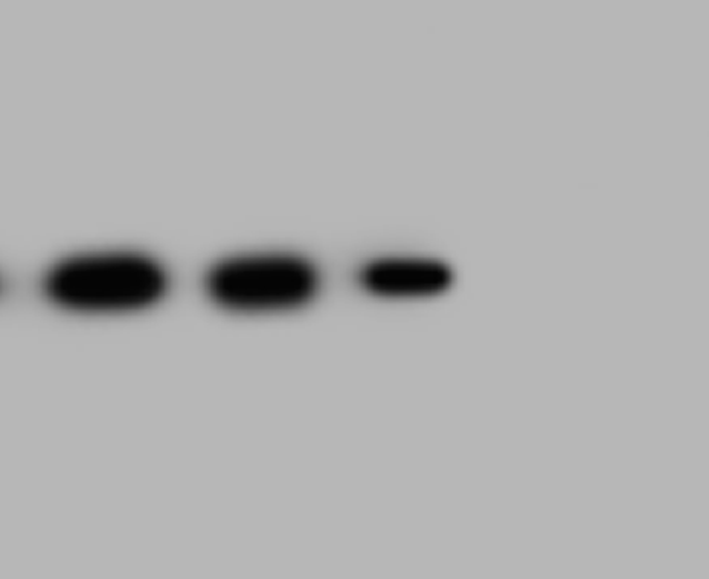

Supplement: Supplemental Material [file KBIE_A_2060626_SM1614.zip › supplementary materials/Original blots of western blot assay/Figure5/p53.tif]

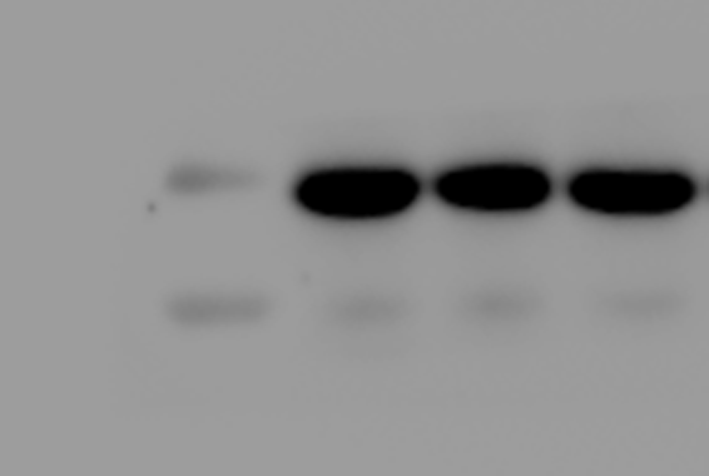

Supplement: Supplemental Material [file KBIE_A_2060626_SM1614.zip › supplementary materials/Original blots of western blot assay/Figure6/GAPDH.tif]

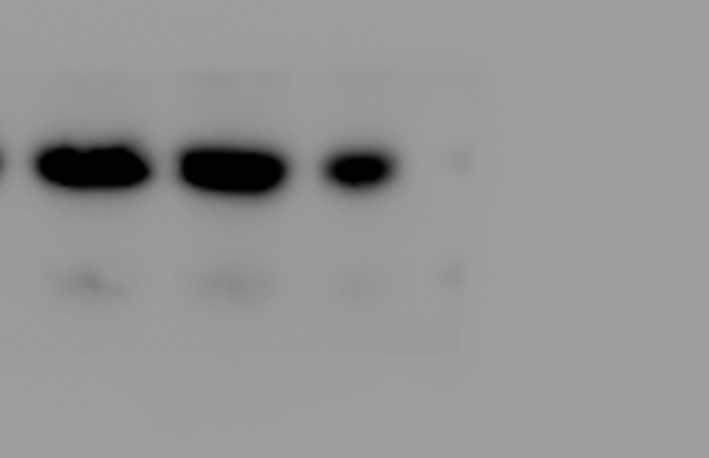

Supplement: Supplemental Material [file KBIE_A_2060626_SM1614.zip › supplementary materials/Original blots of western blot assay/Figure6/SIRT1.tif]

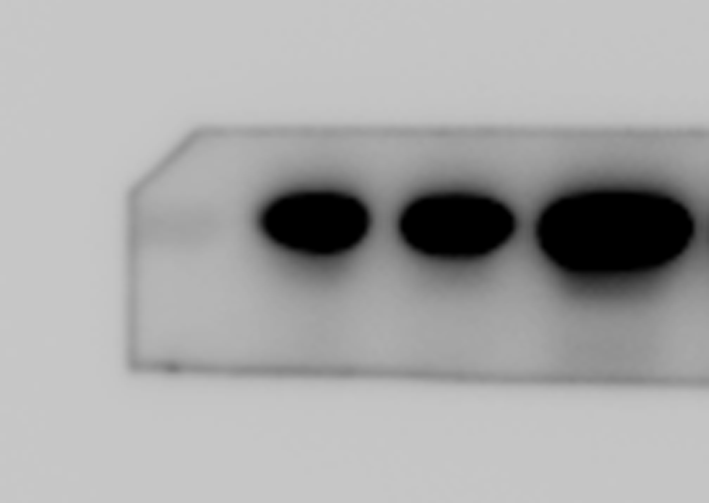

Supplement: Supplemental Material [file KBIE_A_2060626_SM1614.zip › supplementary materials/Original blots of western blot assay/Figure7/Bax.tif]

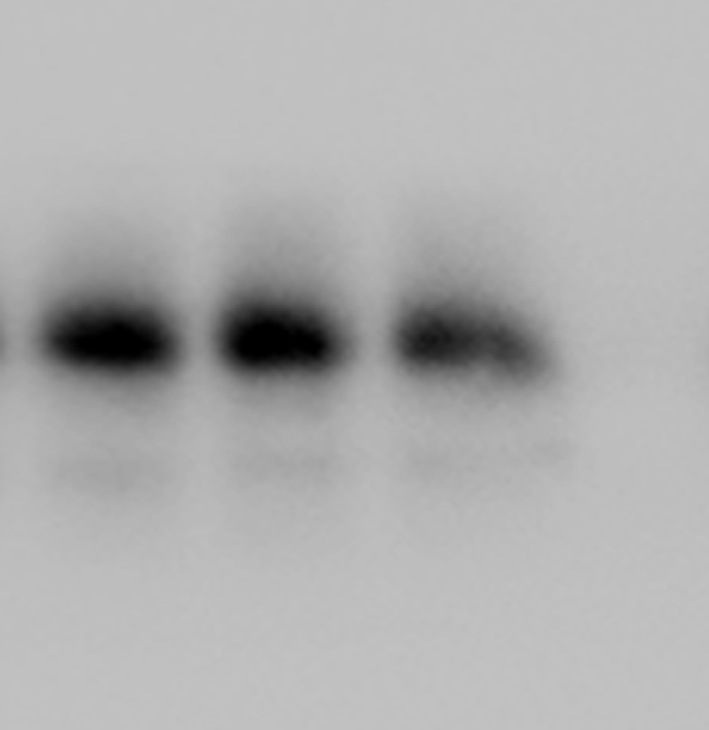

Supplement: Supplemental Material [file KBIE_A_2060626_SM1614.zip › supplementary materials/Original blots of western blot assay/Figure7/Bcl-xl.tif]

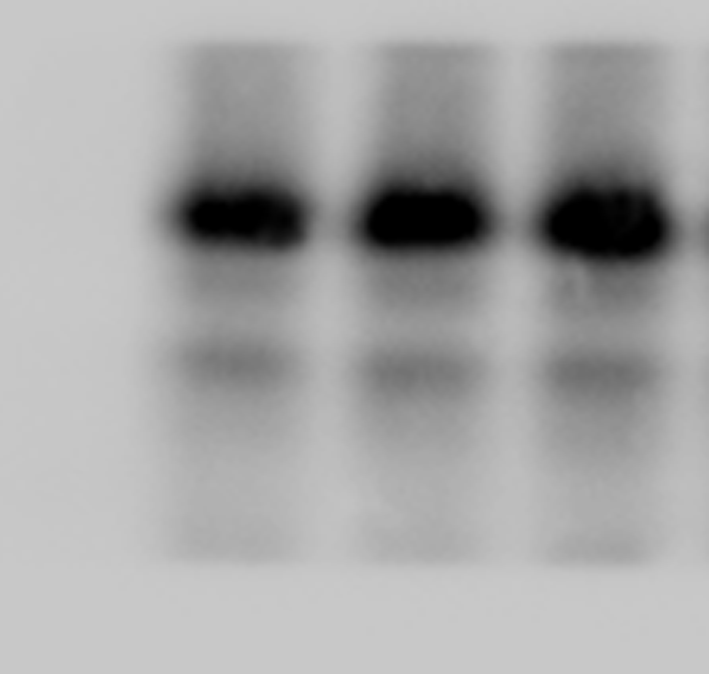

Supplement: Supplemental Material [file KBIE_A_2060626_SM1614.zip › supplementary materials/Original blots of western blot assay/Figure7/GAPDH.tif]

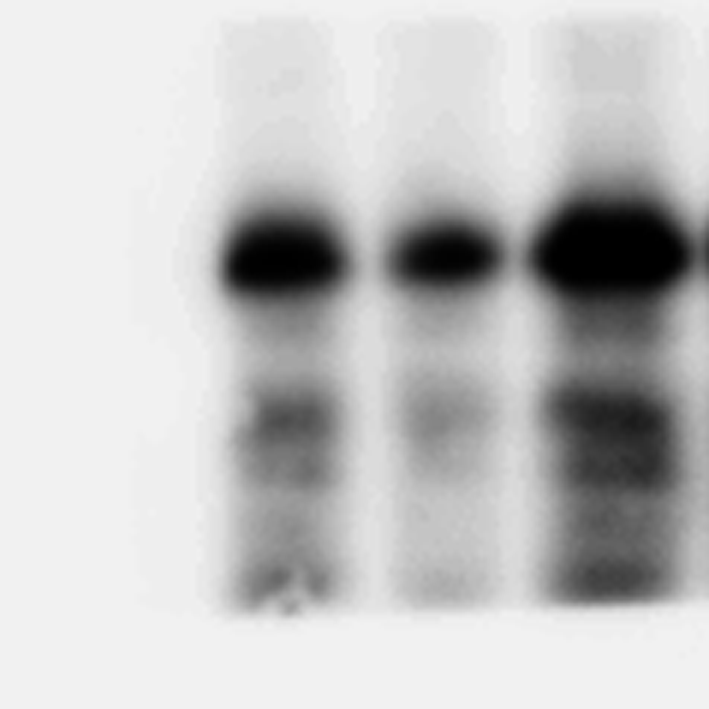

Supplement: Supplemental Material [file KBIE_A_2060626_SM1614.zip › supplementary materials/Original blots of western blot assay/Figure7/cytochrome c.tif]

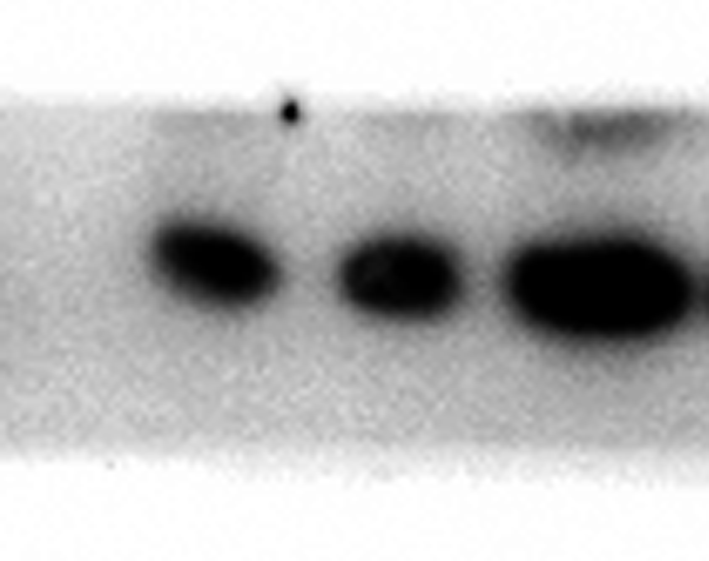

Supplement: Supplemental Material [file KBIE_A_2060626_SM1614.zip › supplementary materials/Original blots of western blot assay/Figure7/p53.tif]

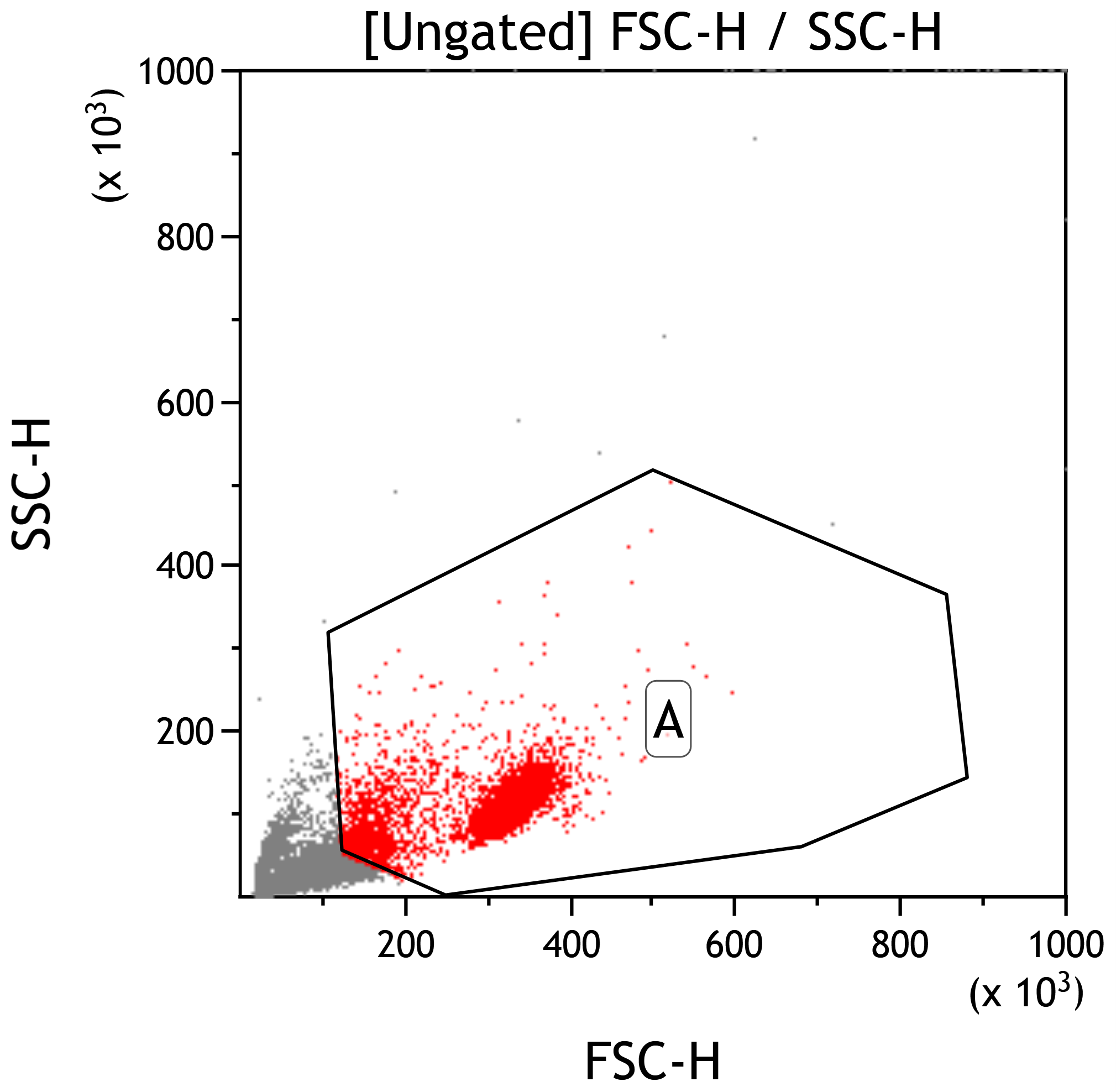

Supplement: Supplemental Material [file KBIE_A_2060626_SM1614.zip › supplementary materials/flow cytometry raw data/Figure 3/CH+SIRT1-plasmid-1.png]

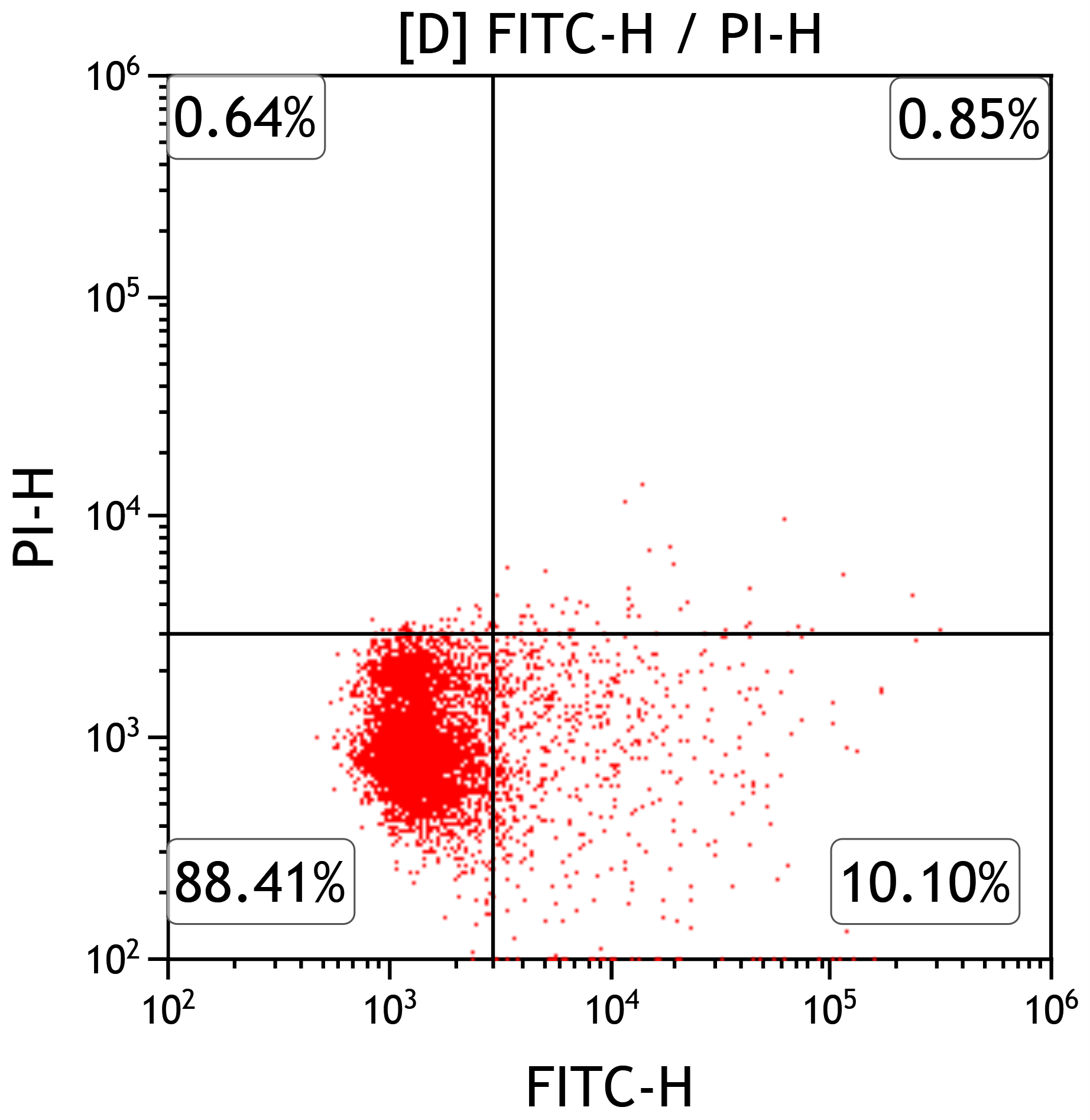

Supplement: Supplemental Material [file KBIE_A_2060626_SM1614.zip › supplementary materials/flow cytometry raw data/Figure 3/CH+SIRT1-plasmid-2.png]

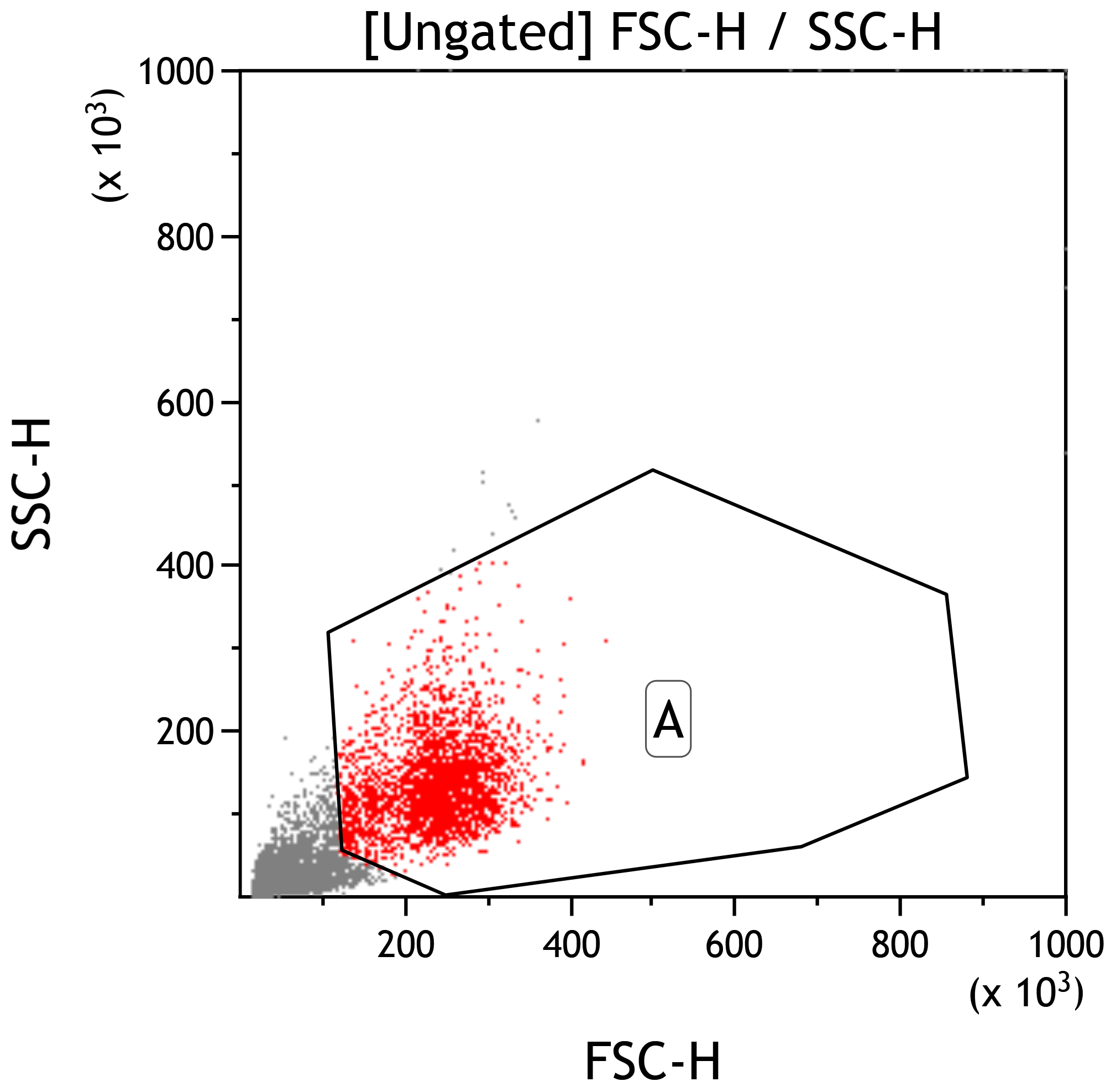

Supplement: Supplemental Material [file KBIE_A_2060626_SM1614.zip › supplementary materials/flow cytometry raw data/Figure 3/CH+control-plasmid-1.png]

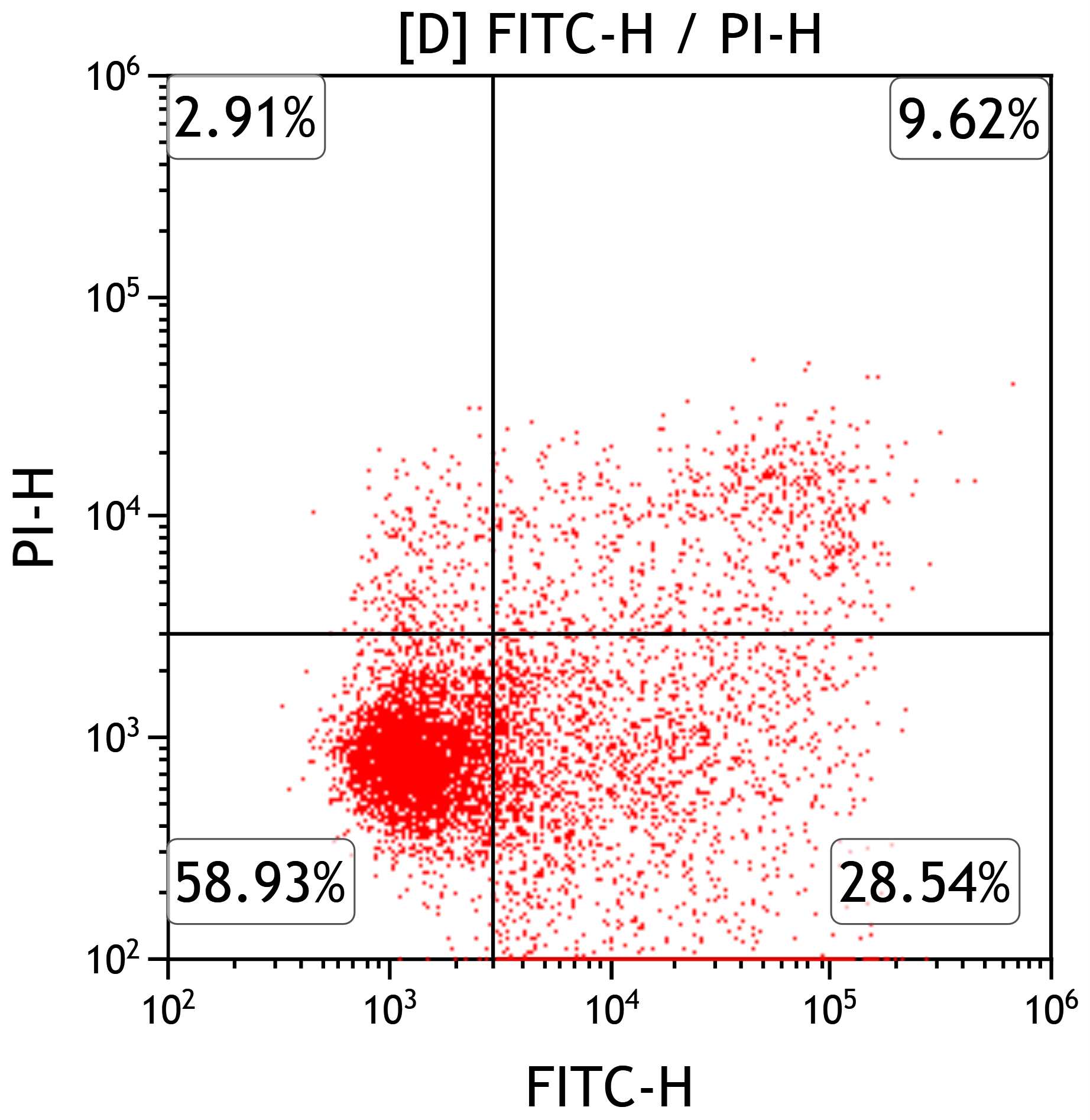

Supplement: Supplemental Material [file KBIE_A_2060626_SM1614.zip › supplementary materials/flow cytometry raw data/Figure 3/CH+control-plasmid-2.png]

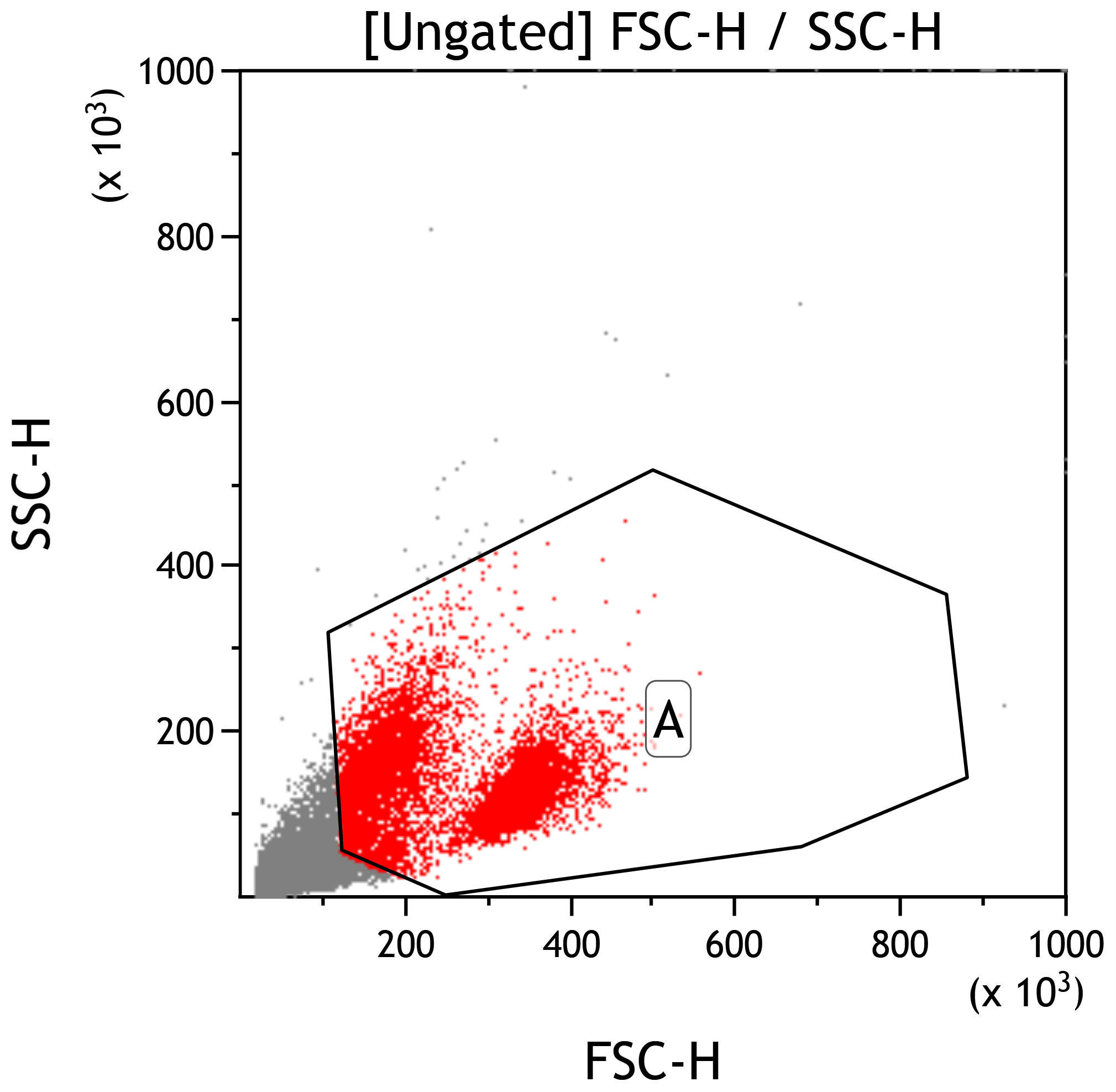

Supplement: Supplemental Material [file KBIE_A_2060626_SM1614.zip › supplementary materials/flow cytometry raw data/Figure 3/CH-1.png]

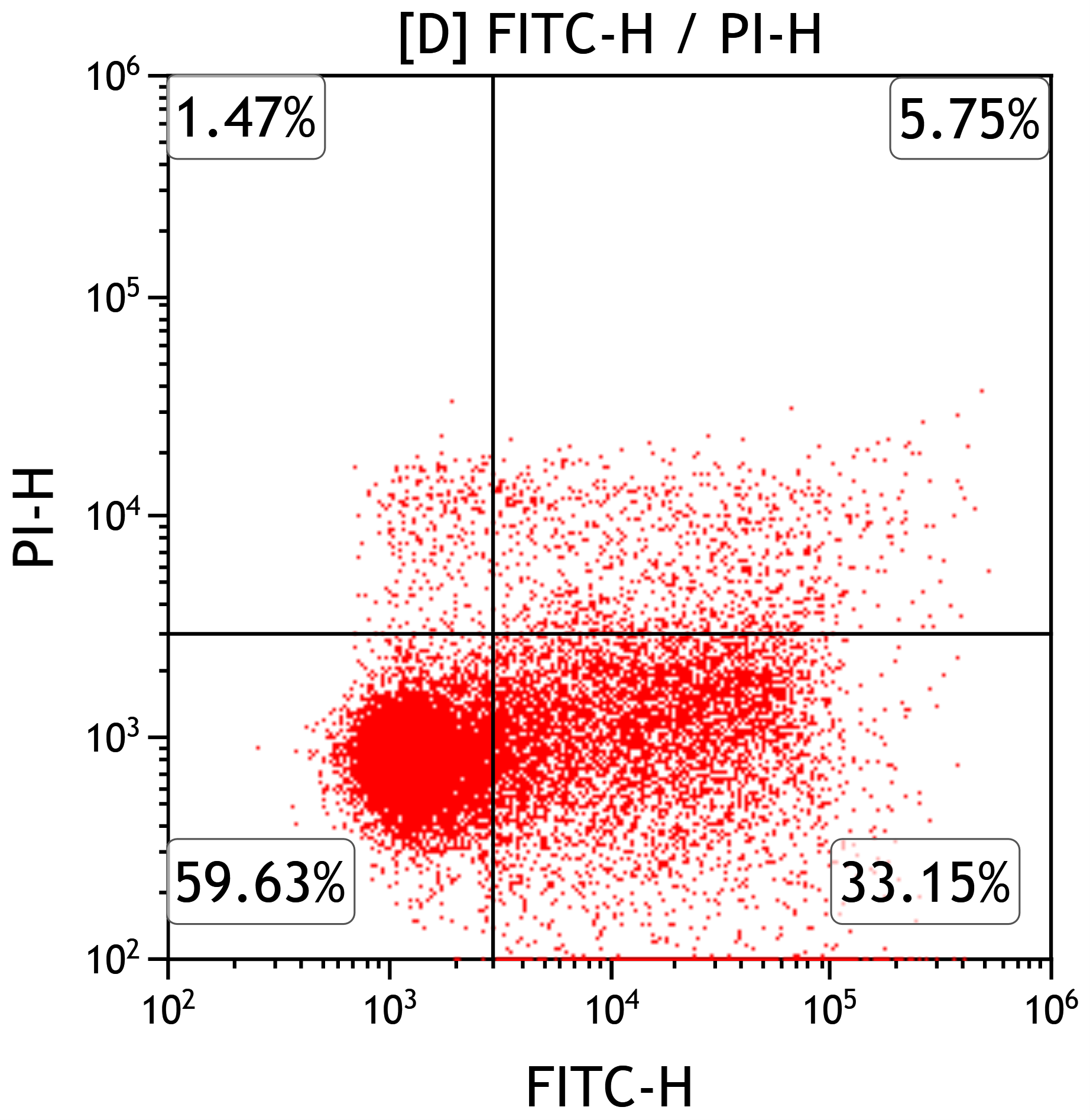

Supplement: Supplemental Material [file KBIE_A_2060626_SM1614.zip › supplementary materials/flow cytometry raw data/Figure 3/CH-2.png]

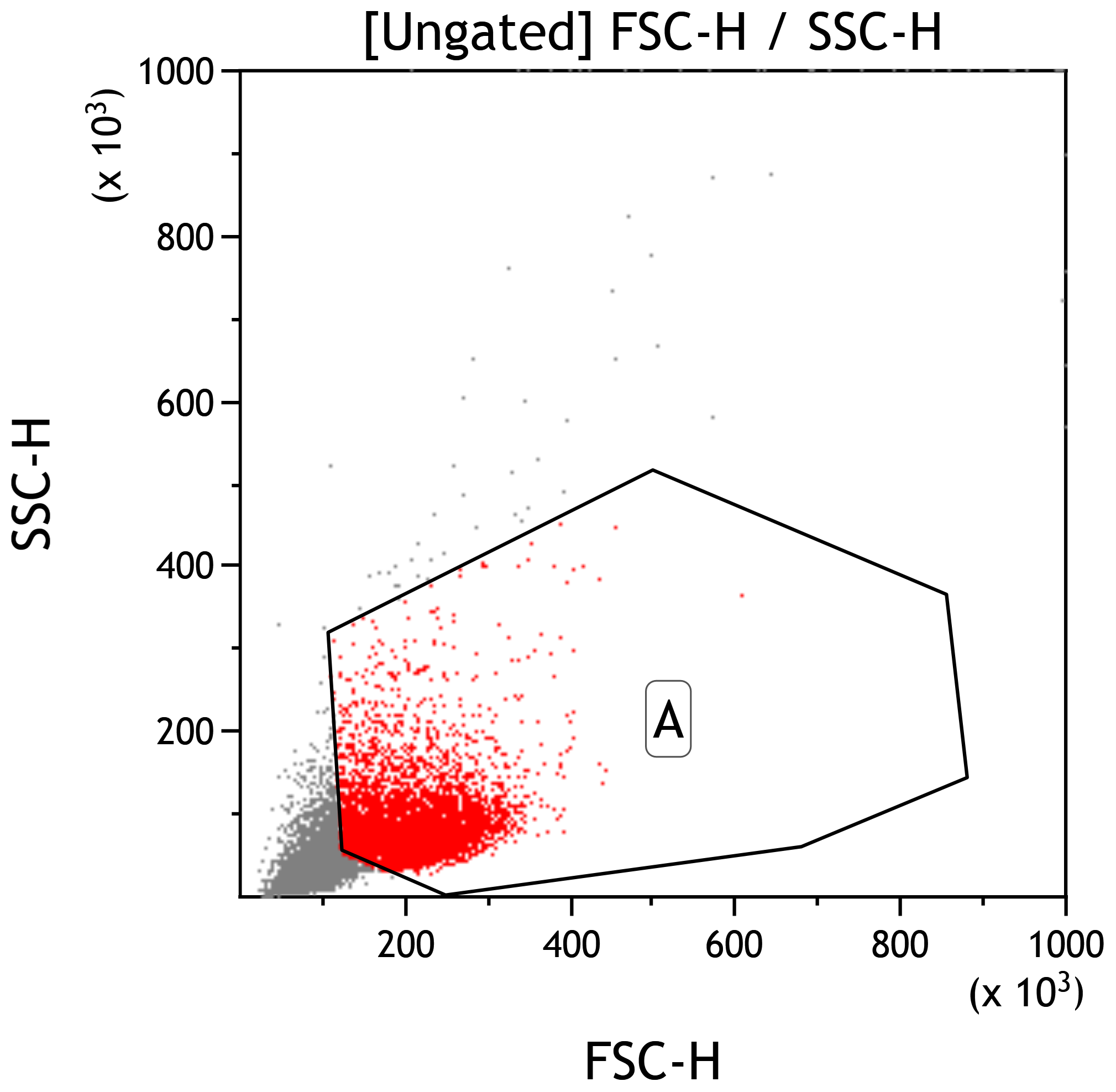

Supplement: Supplemental Material [file KBIE_A_2060626_SM1614.zip › supplementary materials/flow cytometry raw data/Figure 3/Control-1.png]

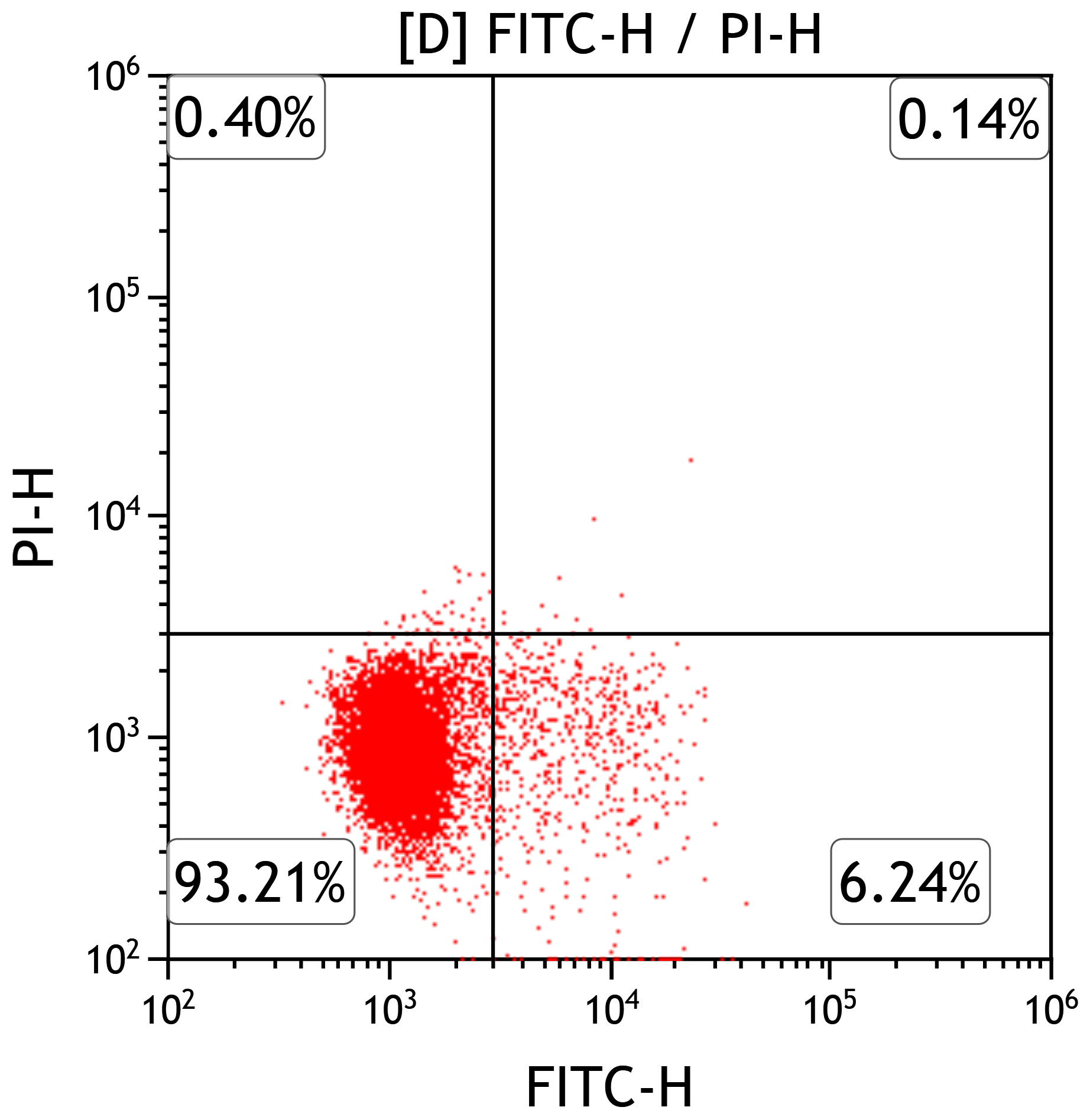

Supplement: Supplemental Material [file KBIE_A_2060626_SM1614.zip › supplementary materials/flow cytometry raw data/Figure 3/Control-2.png]

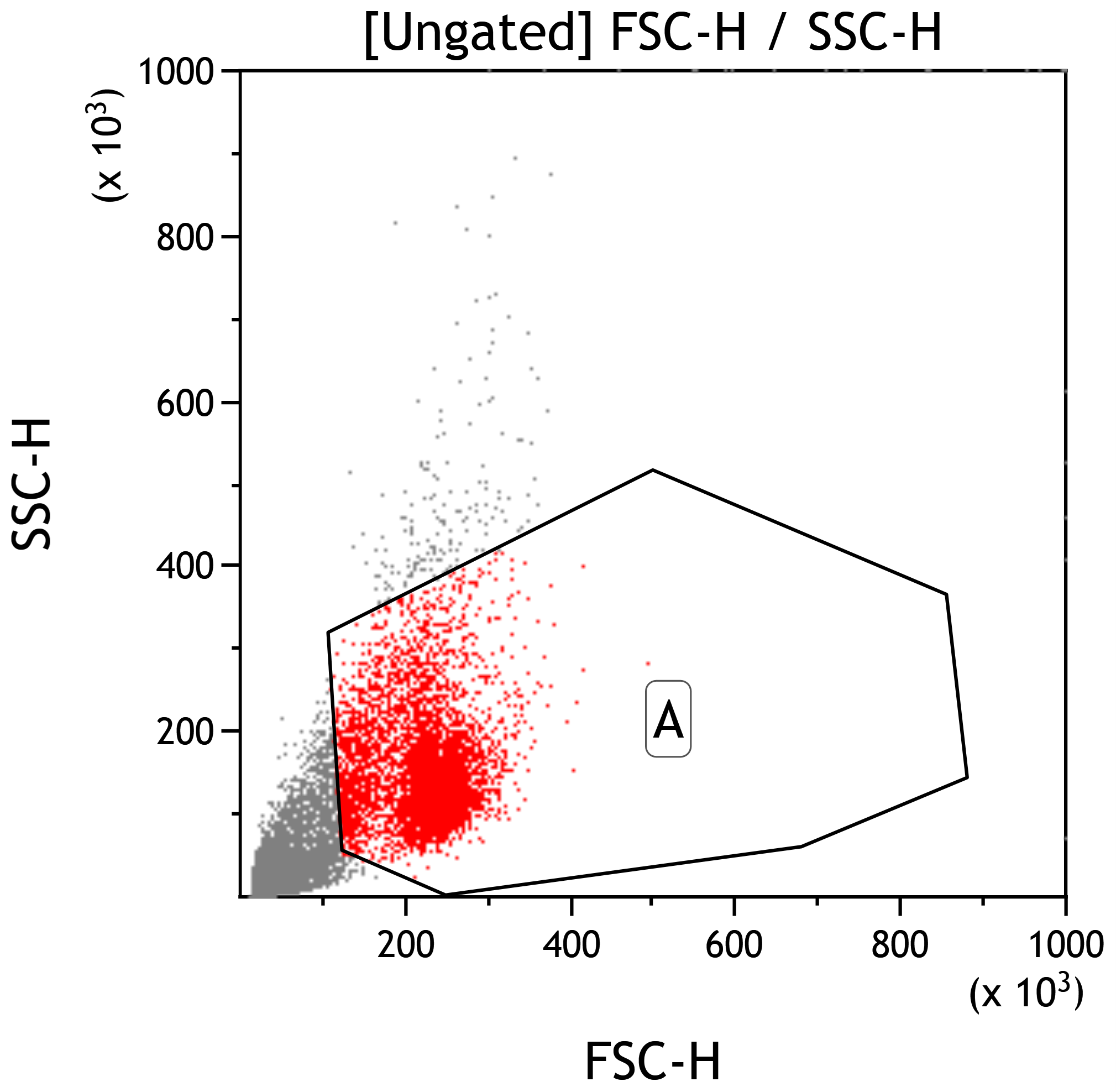

Supplement: Supplemental Material [file KBIE_A_2060626_SM1614.zip › supplementary materials/flow cytometry raw data/Figure 4/Control-1.png]

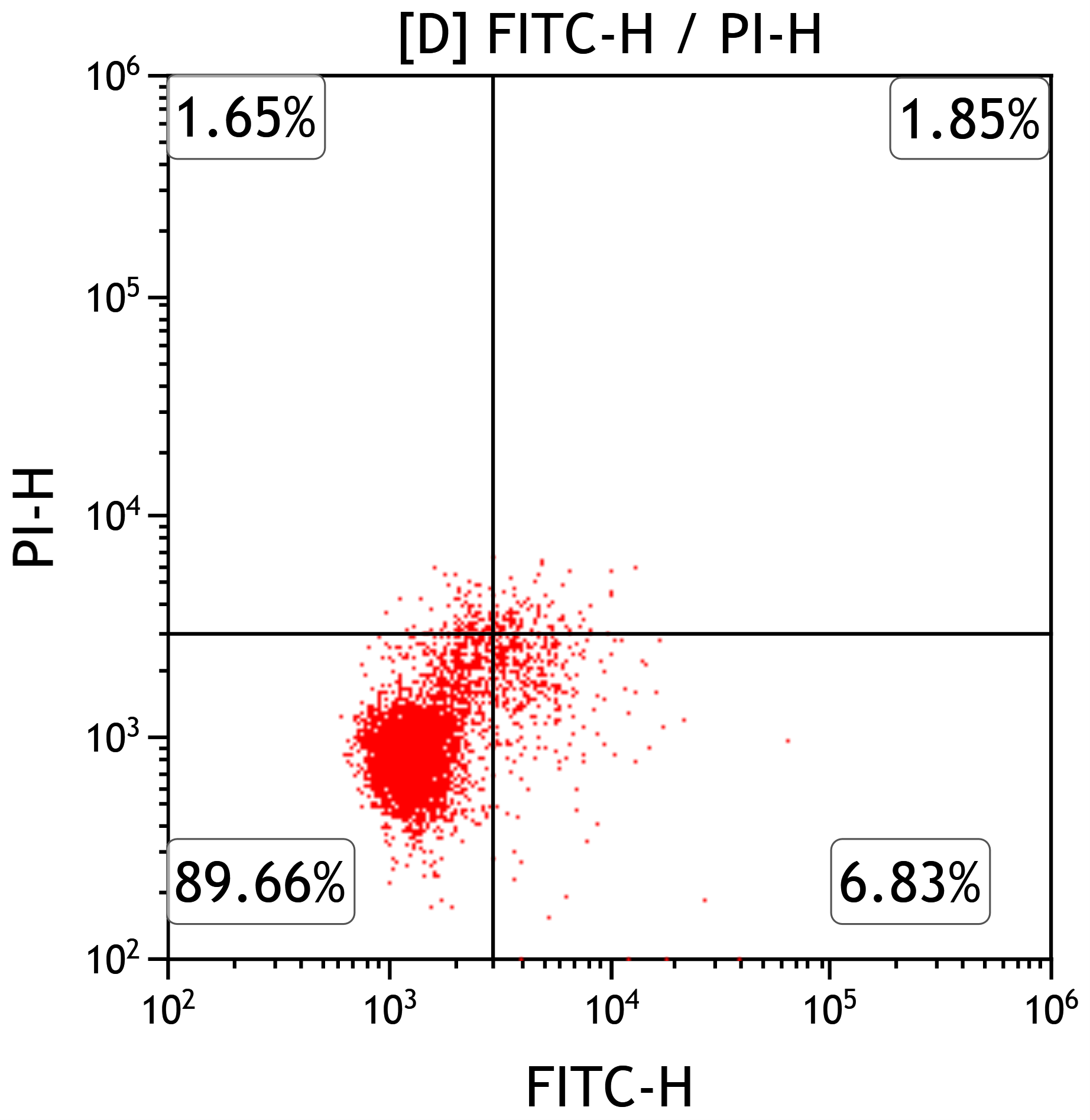

Supplement: Supplemental Material [file KBIE_A_2060626_SM1614.zip › supplementary materials/flow cytometry raw data/Figure 4/Control-2.png]

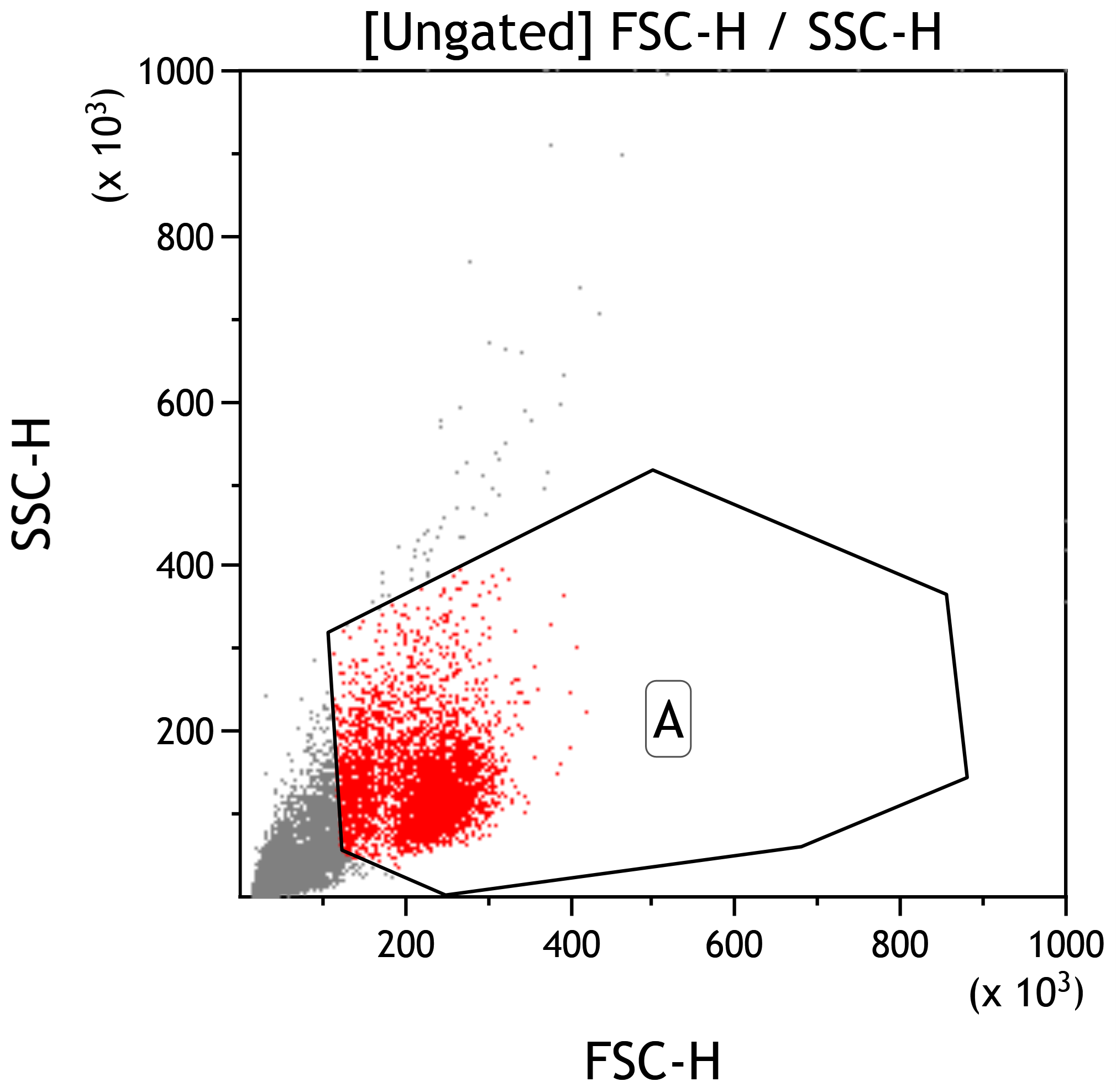

Supplement: Supplemental Material [file KBIE_A_2060626_SM1614.zip › supplementary materials/flow cytometry raw data/Figure 4/Control-plasmid-1.png]

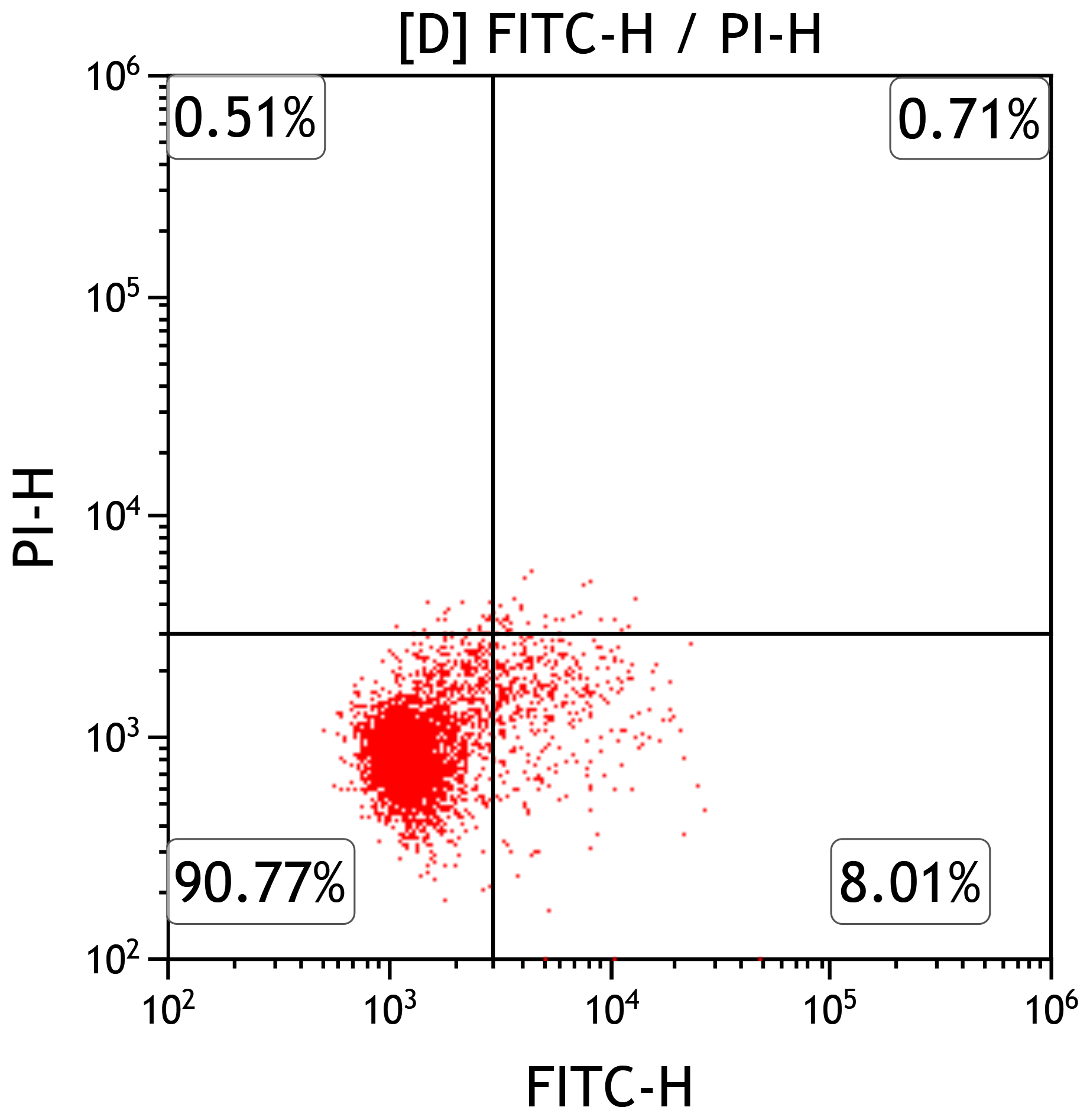

Supplement: Supplemental Material [file KBIE_A_2060626_SM1614.zip › supplementary materials/flow cytometry raw data/Figure 4/Control-plasmid-2.png]

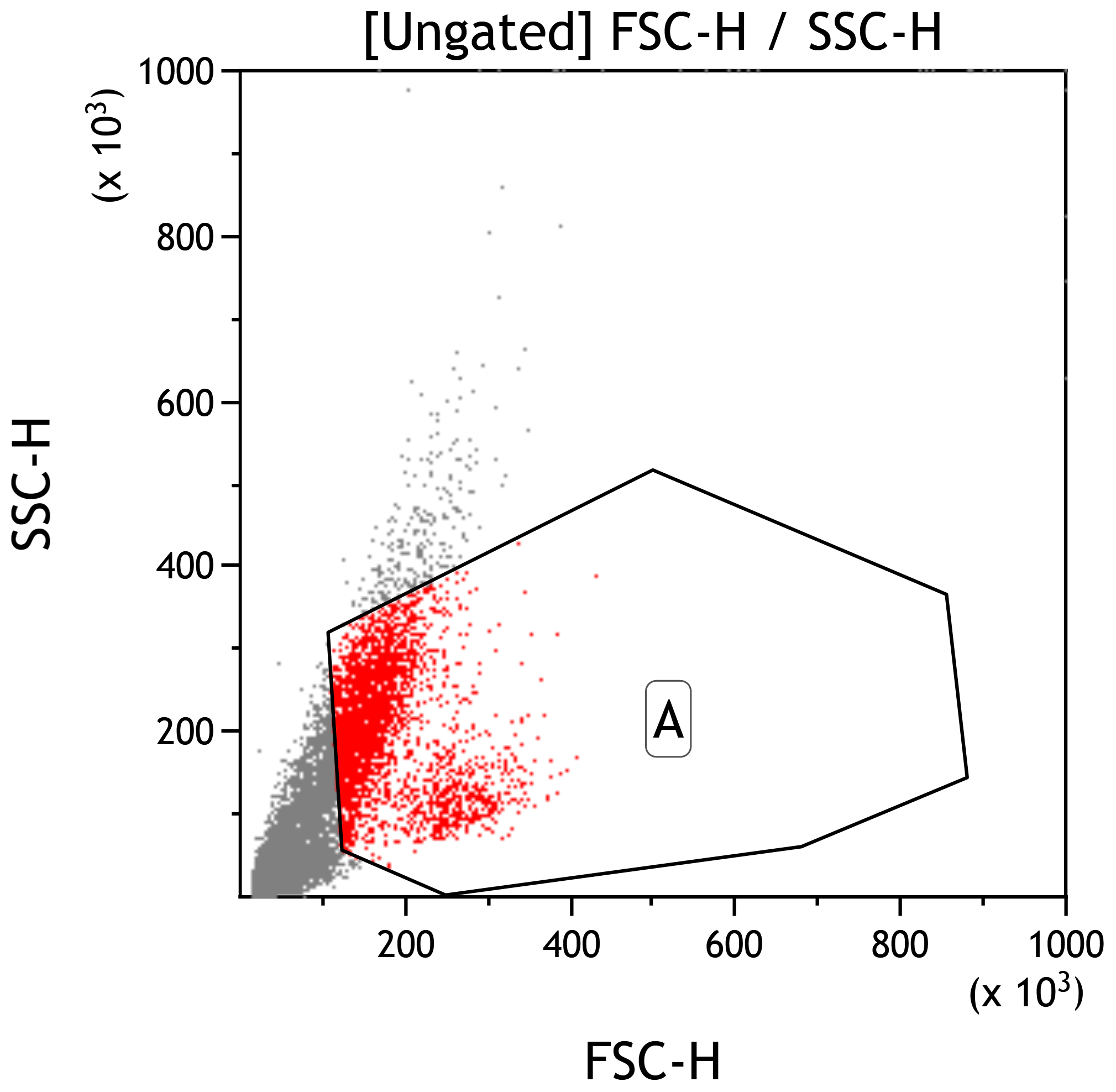

Supplement: Supplemental Material [file KBIE_A_2060626_SM1614.zip › supplementary materials/flow cytometry raw data/Figure 4/SIRT1-plasmid-1.png]

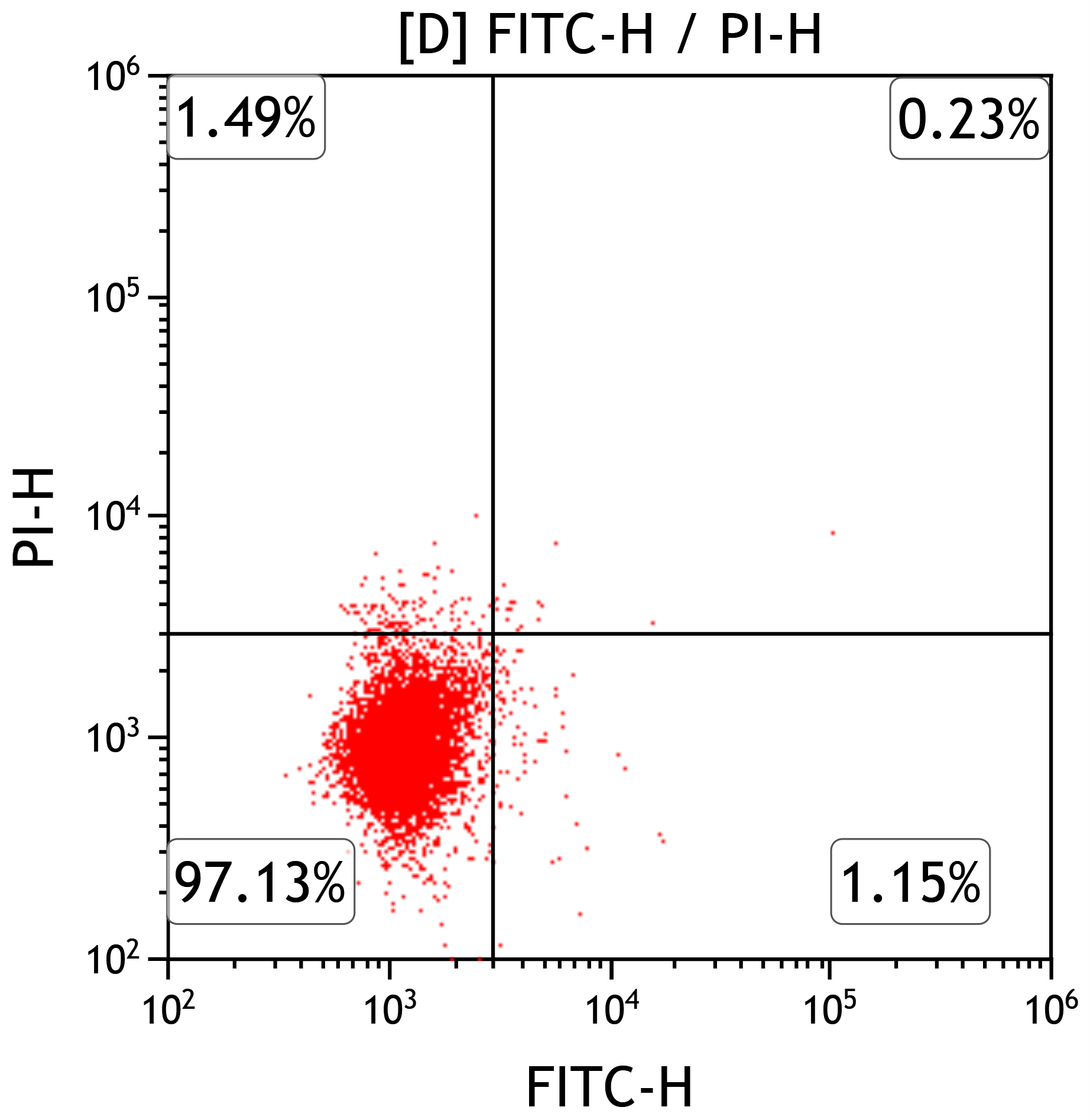

Supplement: Supplemental Material [file KBIE_A_2060626_SM1614.zip › supplementary materials/flow cytometry raw data/Figure 4/SIRT1-plasmid-2.png]

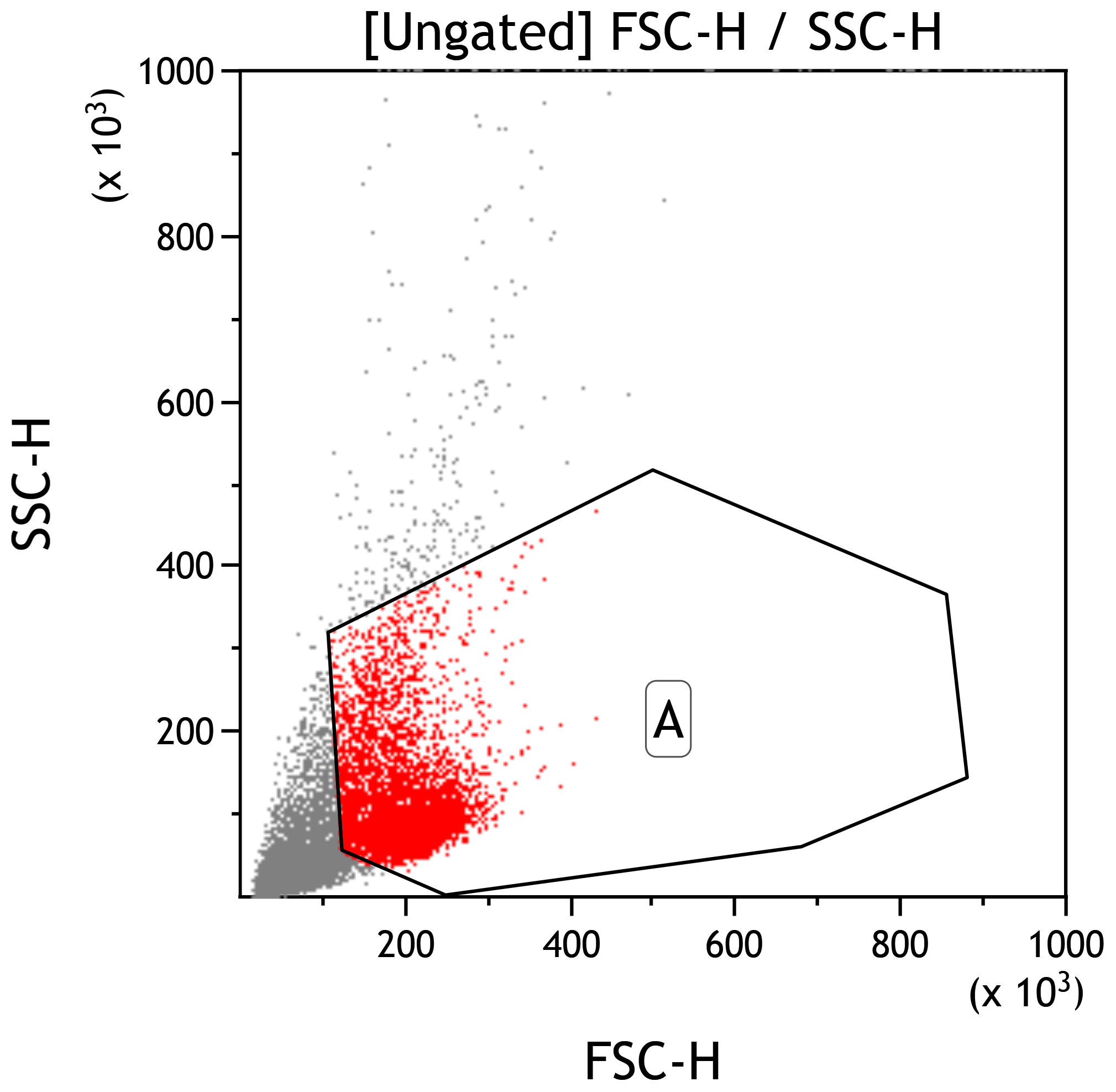

Supplement: Supplemental Material [file KBIE_A_2060626_SM1614.zip › supplementary materials/flow cytometry raw data/Figure 6/Control-1.png]

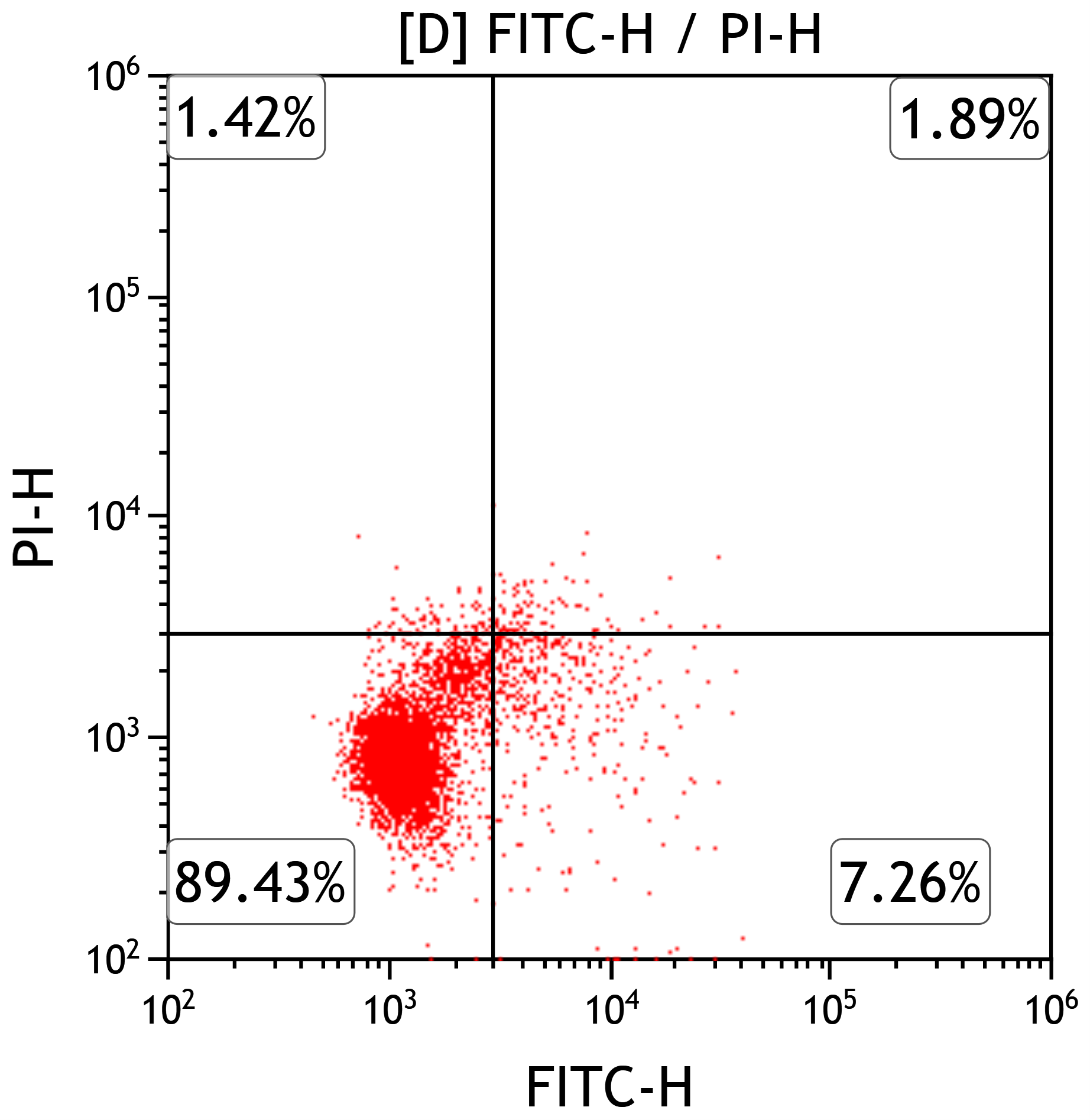

Supplement: Supplemental Material [file KBIE_A_2060626_SM1614.zip › supplementary materials/flow cytometry raw data/Figure 6/Control-2.png]

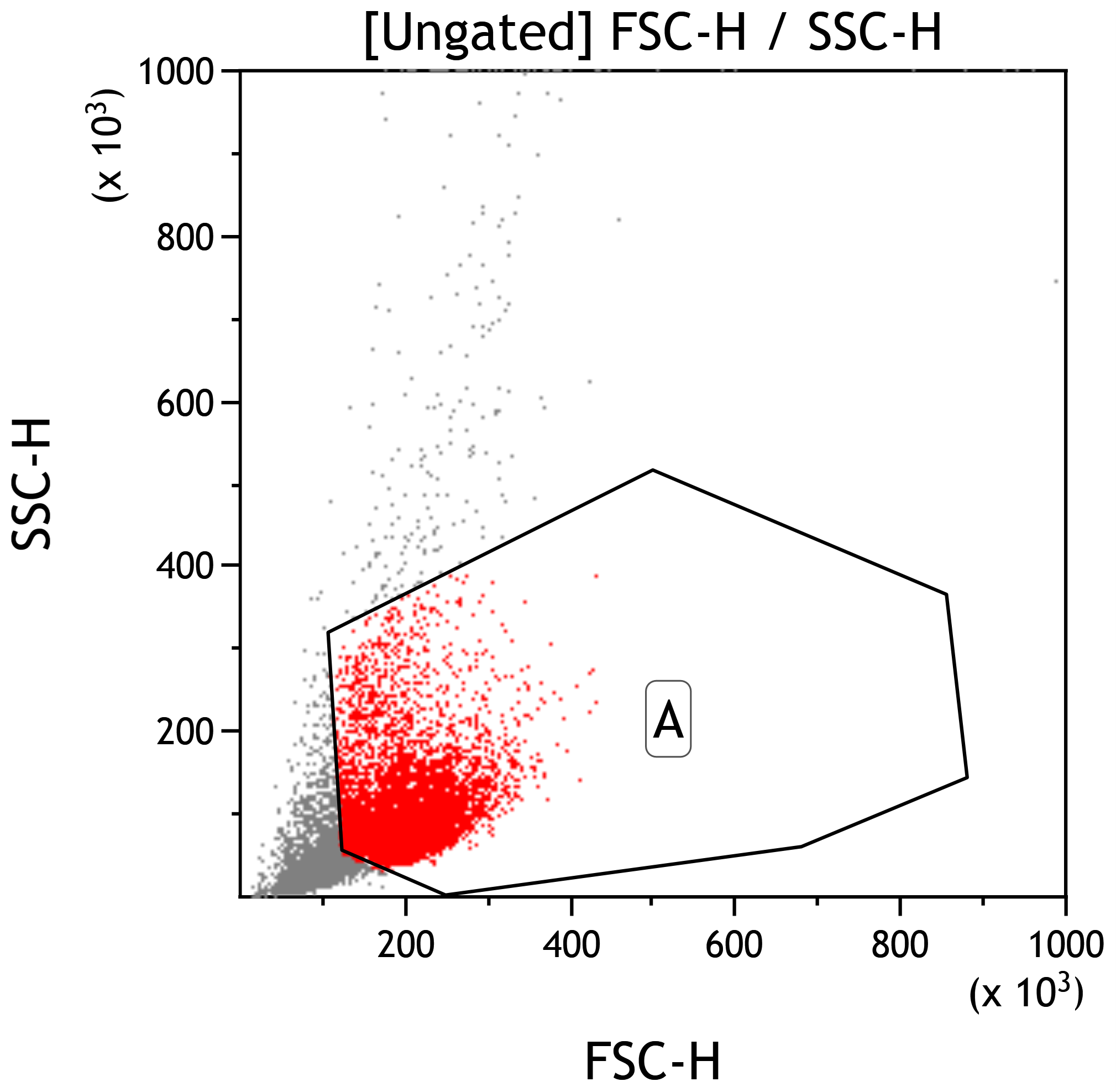

Supplement: Supplemental Material [file KBIE_A_2060626_SM1614.zip › supplementary materials/flow cytometry raw data/Figure 6/Control-siRNA-1.png]

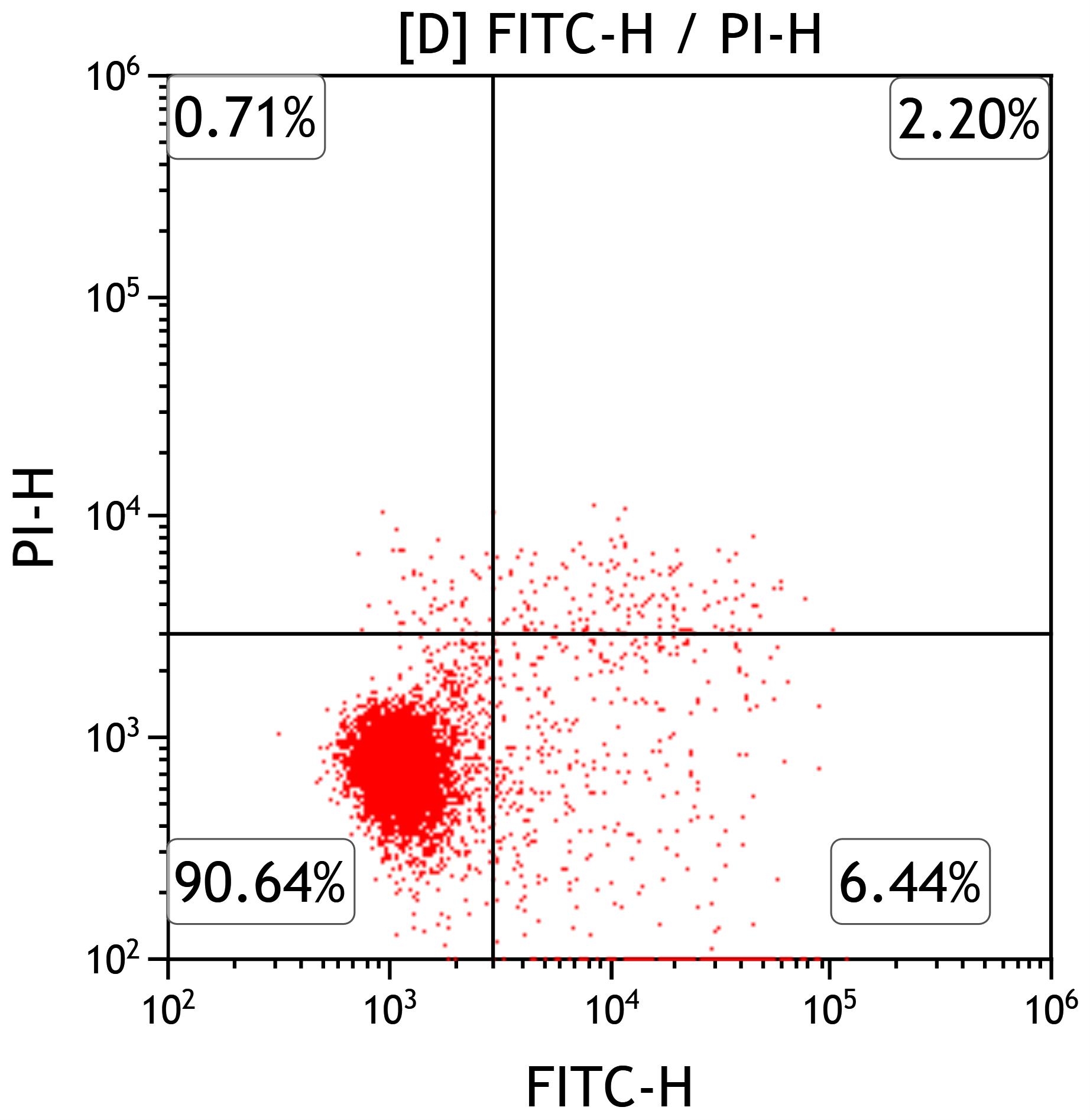

Supplement: Supplemental Material [file KBIE_A_2060626_SM1614.zip › supplementary materials/flow cytometry raw data/Figure 6/Control-siRNA-2.png]

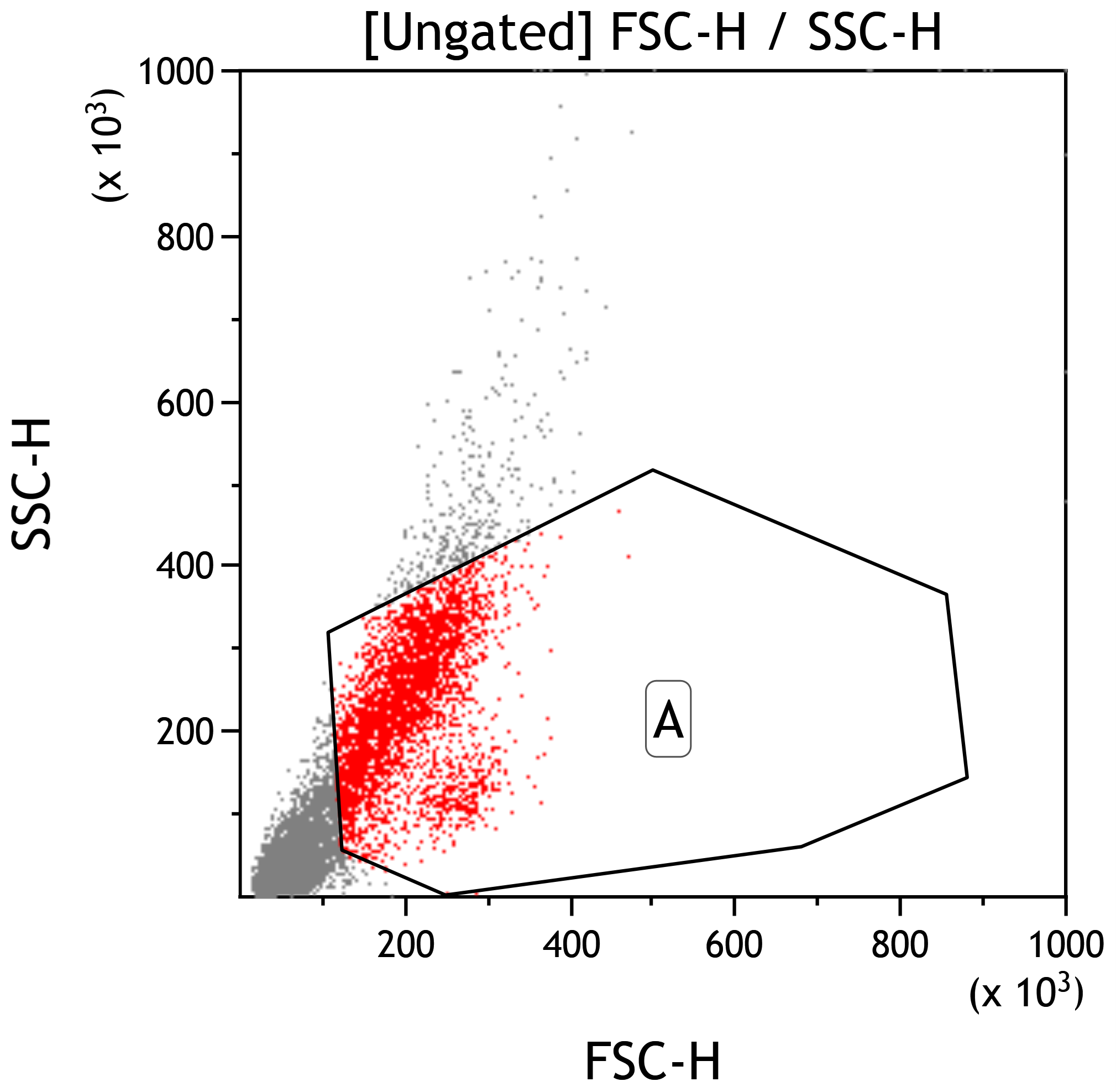

Supplement: Supplemental Material [file KBIE_A_2060626_SM1614.zip › supplementary materials/flow cytometry raw data/Figure 6/SIRT1-siRNA-1.png]

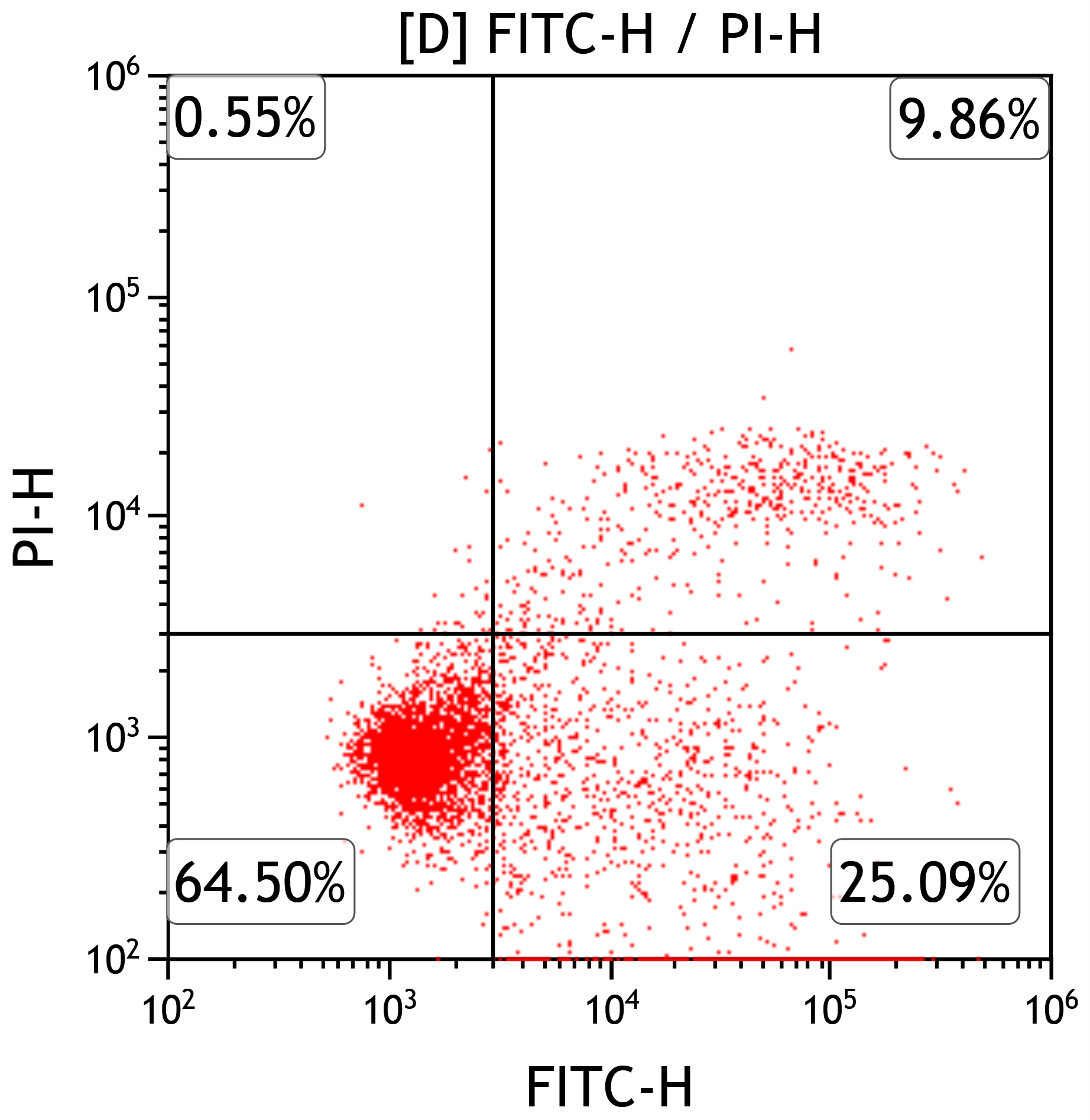

Supplement: Supplemental Material [file KBIE_A_2060626_SM1614.zip › supplementary materials/flow cytometry raw data/Figure 6/SIRT1-siRNA-2.png]
