# Supplementary material for: Role of sirtuin 1 in the brain development in congenital hypothyroidism rats via the regulation of p53 signaling pathway
Source: Bioengineered. 2022 Apr 6;13(4):9455–66. doi: 10.1080/21655979.2022.2060626 (PMC9161855; doi:10.1080/21655979.2022.2060626)
Supplement: Supplemental Material [file KBIE_A_2060626_SM1614.zip › supplementary materials/Original blots of western blot assay/Notes.docx]

Dear editor,

Thank you so much for everything you do for our manuscript. We have provided the original blots as supplementary document for reviewing. And it should be noted that during the Western blot assay, we first cut out the corresponding membrane according to the molecular weight of the target protein and then incubate the primary antibody. Therefore our original imprint is not a full film. We guarantee that all our experiments are carried out scientifically and strictly in accordance with the experimental procedures. Hope to get your understanding and support. We hope this is acceptable, otherwise we will redo this part of the experiment as requested.
